# Highly Cooperative Photoswitching in Dihydropyrene Dimers

**DOI:** 10.1002/anie.202008523

**Published:** 2020-08-26

**Authors:** Pauline Liesfeld, Yves Garmshausen, Simon Budzak, Jonas Becker, André Dallmann, Denis Jacquemin, Stefan Hecht

**Affiliations:** ^1^ Department of Chemistry & IRIS Adlershof Humboldt-Universität zu Berlin Brook-Taylor-Strasse 2 12489 Berlin Germany; ^2^ Department of Chemistry Faculty of Natural Sciences Matej Bel University Tajovkého 40 97401 Banská Bystrica Slovakia; ^3^ CEISAM Lab UMR 6230 Université de Nantes CNRS F-44000 Nantes France; ^4^ DWI—Leibniz Institute for Interactive Materials Forckenbeckstrasse 50 52074 Aachen Germany; ^5^ Institute of Technical and Macromolecular Chemistry RWTH Aachen University Worringer Weg 2 52074 Aachen Germany

**Keywords:** cooperative effects, donor–acceptor systems, photochromism, UV/vis spectroscopy

## Abstract

We present a strategy to achieve highly cooperative photoswitching, where the initial switching event greatly facilitates subsequent switching of the neighboring unit. By linking donor/acceptor substituted dihydropyrenes via suitable π‐conjugated bridges, the quantum yield of the second photochemical ring‐opening process could be enhanced by more than two orders of magnitude as compared to the first ring‐opening. As a result, the intermediate mixed switching state is not detected during photoisomerization although it is formed during the thermal back reaction. Comparing the switching behavior of various dimers, both experimentally and computationally, helped to unravel the crucial role of the bridging moiety connecting both photochromic units. The presented dihydropyrene dimer serves as model system for longer cooperative switching chains, which, in principle, should enable efficient and directional transfer of information along a molecularly defined path. Moreover, our concept allows to enhance the photosensitivity in oligomeric and polymeric systems and materials thereof.

## Graphical abstract

Enhancing photoswitching efficiency: In the donor–acceptor substituted dihydropyrene dimer the quantum yield for the second ring‐opening is enhanced by more than two orders of magnitude compared to the first ring‐opening. Responsible is a cooperative switching effect which has been rationalized by theoretical calculations and is a general phenomenon for dihydropyrene dimers connected through suitable bridges in the 2,7‐positions.

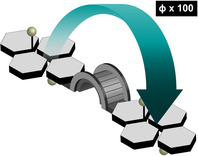

## Introduction

Photochromic compounds can undergo reversible light‐induced isomerization processes, which allow to precisely and conveniently switch between different molecular properties, thus sparking the development of photoresponsive and photoactive materials and devices.^1^ In this context, it is important to integrate not just one but many photochromic units into the desired material, typically by covalent integration, most frequently in the side chain of linear polymers as well as polymer networks.^2^ However, when attempting to improve the response of the individual photochromic units by electronic coupling, for example via π‐conjugated connectors in the main chain, it has proven challenging to retain their switching abilities.^3^ While the large geometrical changes occurring during *E‐Z* photoisomerization of azobenzenes and related photoswitches simply cannot be accommodated in the constraints of a solid matrix, the reasons for largely reduced switching efficiencies of coupled ring‐closing/opening photoswitches, most notably dithienylethenes (DTEs) known to proceed even in the crystalline solid,^4^ appear less obvious. In the majority of systems composed of multiple DTE units,^5^ it has been found that energy transfer to the initially switched, i.e., closed, DTE hinders switching (closure) of another open DTE unit.^6^ In some non‐symmetric dimers both DTEs could be switched individually but not consecutively,^7^ whereas in other DTE dimers subsequent switching was observed yet full conversion to the closed‐closed isomer could not be achieved.6a, 8 There is only one exceptional report of a non‐symmetrical DTE trimer reaching five out of eight possible isomers, and exhibiting four different colors after irradiation with specific wavelengths.^9^ The negative impact of electronic coupling is also apparent from the isomerization behavior of azobenzene dimers^10^ and can only be overcome in the related polymers by decoupling the individual azobenzene units in the main chain.^11^

Interestingly, the use of *trans*‐15,16‐dimethyl‐15,16‐dihydropyrene (DHP), first synthesized by Boekelheide and co‐workers^12^ and further developed as a photoswitch by the group of Mitchell,^13^ can overcome these problems completely as described below. In fact, Mitchell and co‐workers were the first to report two cases of functional three‐state photoswitches, in which both DHP units open and close.^14^ The same group could go even one step further by fusing three DHP units and despite the rather short thermal lifetime of the photogenerated cyclophanediene (CPD) they were able to detect four out of six possible switching states.^15^ The DHP‐CPD system therefore seems to be particularly suitable for multiphotochromic systems since the switching ability is not adversely affected when multiple DHPs are π‐conjugated.^16^ Moreover, thanks to insights from both experiment and theory, the rather complicated mechanism of DHP photoisomerization has been elucidated to a great extent.^17^ In the parent DHP, excitation causes population of the second singlet excited state (*S_2_*) possessing zwitterionic character. From there, however, deactivation through a conical intersection to the non‐photoswitching *S_1_* is highly efficient and is responsible for the low quantum yield of DHP ring‐opening (Φ_c→o_=0.02 in ethanol^18^ and Φ_c→o_=0.006 in cyclohexane^19^). This non‐productive deactivation pathway can be circumvented by introducing proper substituents,^20^ in particular in positions 2 and 7 along the long axis of the molecule.^21^ We have recently investigated the effect of “push‐pull” functionalization for both the 2,7‐ and 4,9‐substitution patterns and found that donor‐acceptor substitution enhances the photochemical ring‐opening efficiency.^22^

**Figure 1:**
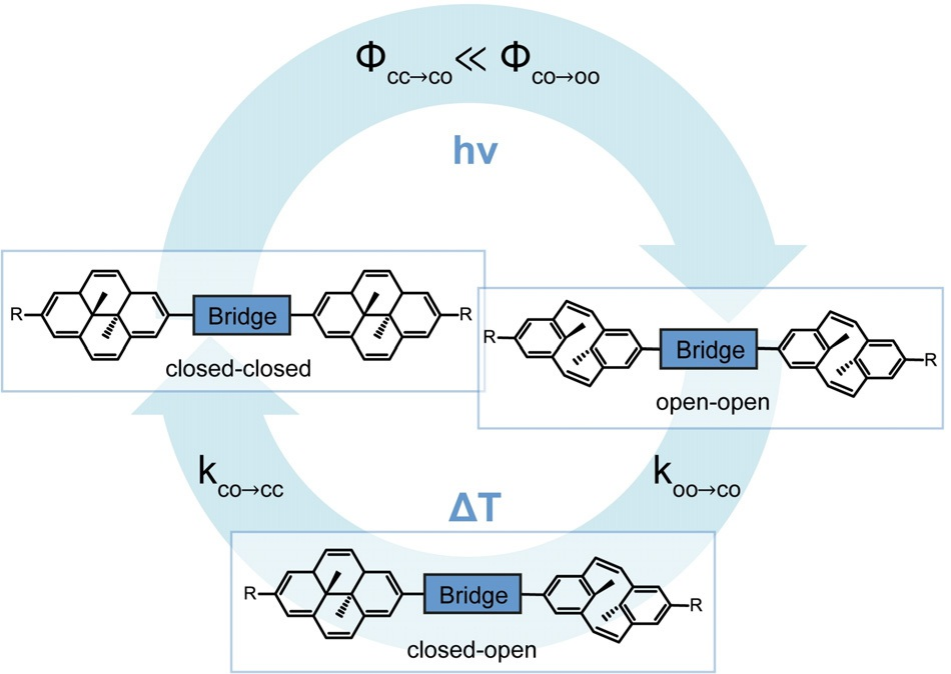
Concept of cooperative switching in DHP chains, shown for the simplest case of a DHP dimer, based on connecting two DHPs via a π‐conjugated bridge. Since the quantum yield for the second photochemical ring‐opening is much higher, the intermediate is not observed during irradiation, whereas it is built up and thus observed during the thermal back reaction.

Based on this knowledge and our most recent findings, we have been able to realize pronounced cooperative switching in DHP dimers. Our working principle relies on alternating electron donating and accepting character on opposing DHP termini. Illustrating this for the symmetrical dimer case (Figure 1), the bridge is serving as electron donor (or acceptor) to *both* DHP units, which carry terminal electron accepting (or donating) groups. Upon ring‐opening of the first DHP unit, the extended π‐conjugation of the closed‐closed (cc) form is broken and the donating ability of the bridge moiety to the second DHP unit is enhanced, thereby largely increasing the efficiency for the second ring‐opening event. This leads to a cooperative switching phenomenon, which manifests itself by the absence of any notable amount of closed‐open (co) intermediate during photoisomerization.^23^ However, since the individual rates of the thermal ring‐closure events do not exhibit such large variation, the closed‐open species is being built up and consequently observed in the course of the thermal back reaction. Here, we present our comprehensive experimental and theoretical work on several DHP dimers and disclose it as a general strategy to achieve highly cooperative switching in linear π‐conjugated multi‐switch chains.

## Results and Discussion

**Figure 2:**
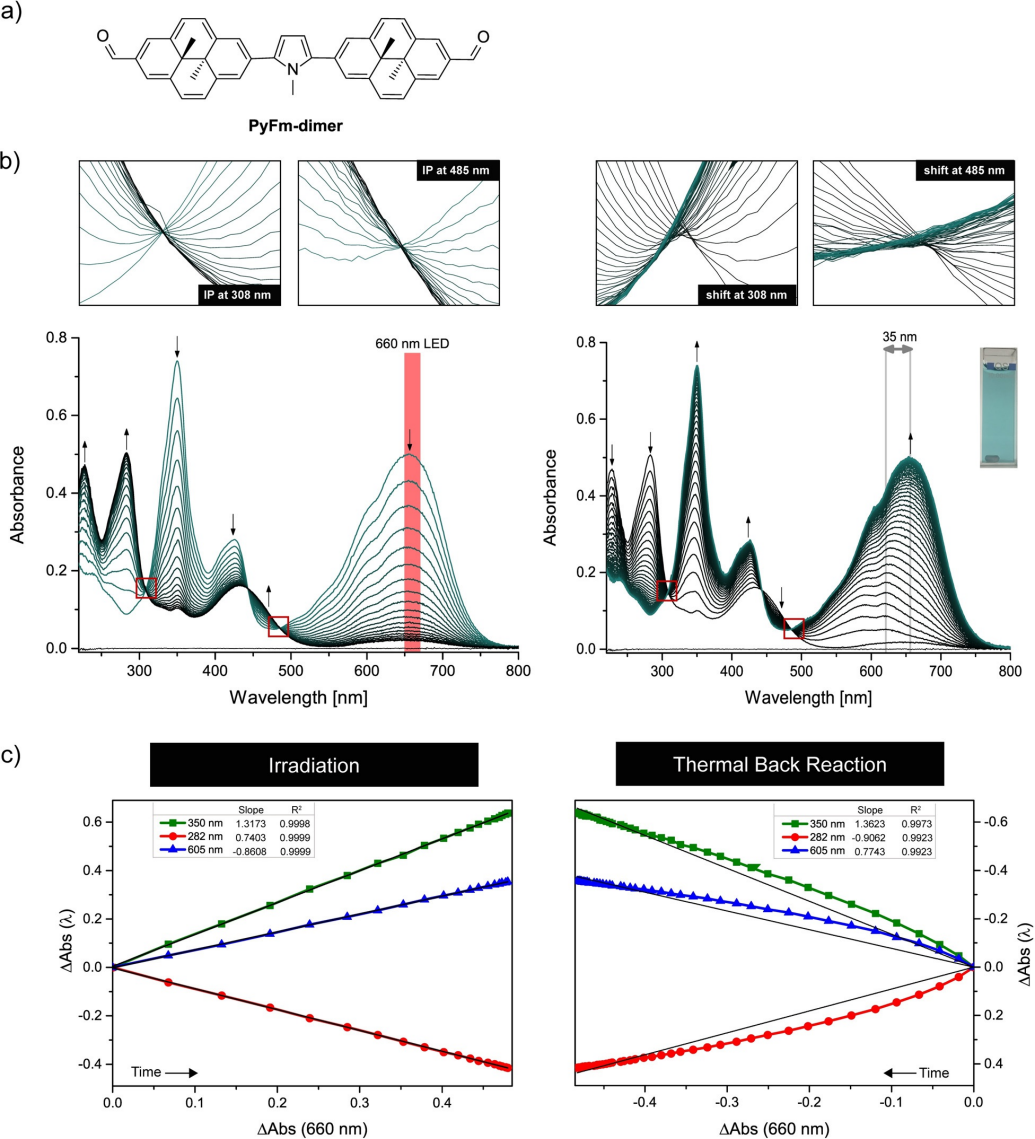
a) Chemical structure of the investigated **PyFm‐dimer**. b) UV/vis spectral changes upon irradiation with a 660 nm LED (left, 1 min between consecutive spectra) and during thermal back reaction (right, 2 min between consecutive spectra) of **PyFm‐dimer** at 0 °C in THF (7.5×10^−6^ M). Insets above show the presence or absence of isosbestic points (IP) in enlarged spectral regions. c) Respective absorbance difference diagrams.

The molecular design of the representative **PyFm‐dimer** (Figure 2 a) involves *N*‐methylpyrrole as the bridge because it has a moderate donating character and the α‐positions offer a feasible way for bis‐functionalization via CH‐activation. Electron‐withdrawing formyl groups were attached on the other DHP termini to install the desired push‐pull character. Details about the synthesis including experimental procedures and characterization data are available in the Supporting Information.

The **PyFm‐dimer** shows a strong absorption maximum in the red region of the spectrum. When irradiated with a 660 nm LED the band gradually decreases with no observable shift, while two new bands appear in the UV region (Figure 2 b, left). Clean isosbestic points are present, indicating an *immediate* conversion from the closed‐closed form to the open‐open (oo) isomer (Figure 2 b left, insets). During the thermal back reaction there is an initial shift of 35 nm to lower wavelengths before the initial spectrum of the closed‐closed isomer is recovered (Figure 2 b, right). The isosbestic points vanished, supporting the built‐up of the intermediate closed‐open form (Figure 2 b right, insets). Using absorbance difference diagrams developed by Mauser^24^ that show linearity for the photoisomerization process (Figure 2 c, left), we infer an immediate conversion of the closed‐open to the open‐open form, indicating a much higher quantum yield for the second ring‐opening as compared to the first one. Importantly, the same analysis shows strong deviation from linearity for the thermal ring‐closure (Figure 2 c, right), in line with the build‐up of a significant concentration of the intermediate closed‐open isomer. Note that due to the donor‐acceptor substitution the thermal back reaction is relatively fast and thus both irradiation and thermal back reaction as described above were carried out at 0 °C to allow for more convenient monitoring. Importantly, no differences were observed between irradiating degassed and aerated solutions, demonstrating that **PyFm‐dimer** is not prone to endoperoxide formation common in case of many DHP derivatives.^25^

**Figure 3:**
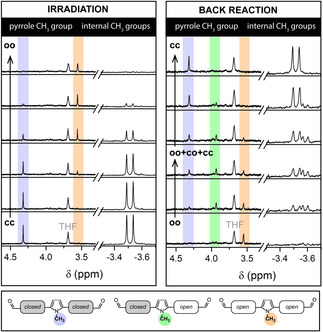
NMR‐spectra during irradiation with a 660 nm LED (left) and thermal back reaction (right) at 15 °C in CD_2_Cl_2_. Shown are the characteristic chemical shifts of the bridge's *N*‐methyl groups and the DHPs’ methyl groups.

These results from UV/vis absorption spectroscopy have been complemented by irradiation experiments carried out directly in an NMR spectrometer using a LED coupled to an optical fiber reaching into the NMR tube. The characteristic signal shifts of the different methyl groups are indicative, on the one hand, of the switching state of the DHP (showing at negative ppm values due to their location inside the aromatic 14π‐electron system of the DHP but not CPD) and, on the other hand, of the electronic nature of the central pyrrole bridge (being coupled to either two, one or no acceptor‐substituted DHP). The irradiation causes the presence of two symmetrical compounds (Figure 3, left) confirming that the closed‐open intermediate is not build up in detectable concentrations during the irradiation because the second photoisomerization step is much more efficient. In strong contrast, the non‐symmetrical closed‐open intermediate can be detected during the thermal back reaction (Figure 3, right). A more detailed assignment of all protons for each isomer is provided in Figures S27 and S28.

**Figure 4:**
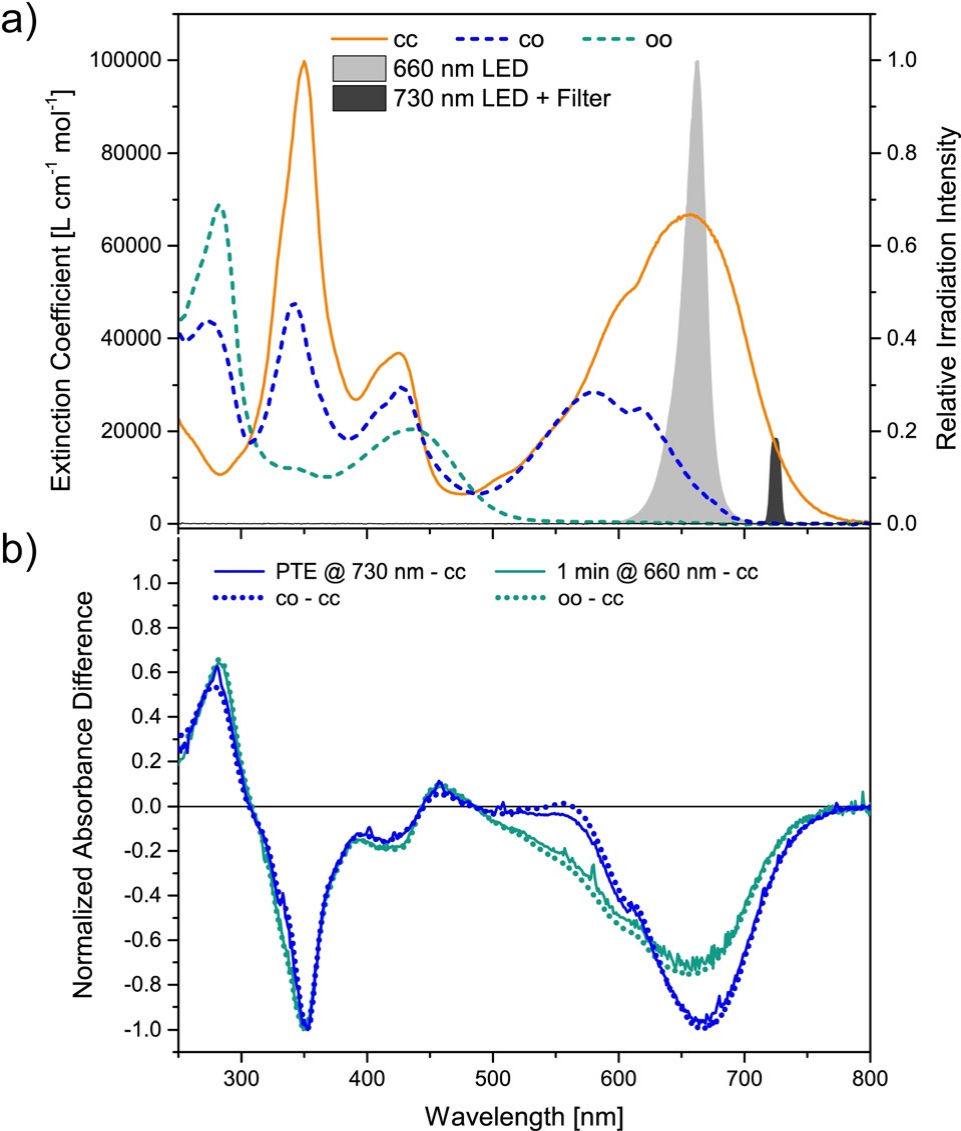
a) Absorption spectra of all three isomers of the **PyFm‐dimer**. While the spectrum of the closed‐closed isomer has been measured, the ones for the closed‐open and open‐open isomers have been derived from the calculated concentrations (see Figure S31). b) Differential spectra obtained during irradiation experiments using either 660 nm (270 mW, for 1 min) or 730 nm (50 mW, until reaching the photothermal equilibrium (PTE)) excitation (solid lines), showing the presence of the open‐open and closed‐open isomer, respectively, by matching the calculated spectra (dotted lines). The spectral profile and relative intensity of the utilized light sources are shown in a).

In order to quantify the extent of cooperativity, the quantum yields for both individual ring‐opening steps were determined. Typically, the thermal instability of T‐type photoswitches complicates such calculation but in this case, it enables the determination of the pure closed‐open spectra since the thermal back isomerization follows reaction kinetics for an irreversible consecutive reaction. After irradiating the **PyFm‐dimer** with 660 nm at 0 °C to the photothermal equilibrium,^26^ the thermal back reaction was monitored at 0 °C in the dark for 60 min (see Figure S29). In the wavelength region of 700–730 nm only the closed‐closed isomer absorbs, allowing calculation of the concentration over time (see Figure S30). By fitting the time‐dependent concentration the rate constants of the first and second thermal ring‐closure at 0 °C could be derived as *k*_oo→co_=8.42±0.04×10^−2^ min^−1^ and *k*_co→cc_=3.82±0.01×10^−2^ min^−1^, respectively (see Figure S31). Thus, the first thermal ring‐closure is roughly twice as fast as the second ring‐closure because the impact of the donor‐acceptor substitution is changed after the first DHP has been reformed and conjugation thus been reestablished. From these values, the concentrations of all isomers during the thermal back reaction were calculated (see Figure S32) and the spectra of pure closed‐open and open‐open isomers could be derived (Figure 4 a).

The closed‐open spectrum resembles the one of the **PyFm‐monomer** (see Figure S35) due to their structural similarity. After obtaining the time‐dependent concentrations of all three isomers during irradiation, the individual quantum yields could be determined as Φ_cc→co_=0.05×10^−2^ and Φ_co→oo_=5.56×10^−2^ based on the least square fit (see Figure S34). The latter value is of the same order of magnitude as the one for the structurally similar **PyFm‐monomer** (see Section 5.2 of the Supporting Information). Most importantly, our findings prove that the efficiency for the second ring‐opening step is enhanced more than 100‐fold as compared to the first one demonstrating large cooperativity of these successive photoswitching events.

Inspection of the measured and derived absorption spectra of all three isomers (Figure 4 a) shows that due to the diminished extent of π‐conjugation in the closed‐open isomer, selective excitation of the closed‐closed isomer above 700 nm should be feasible and thus photoisomerization should be limited to the first ring‐opening reaction. Indeed, when the closed‐closed isomer was irradiated with a 730 nm LED in combination with a narrow 730 nm bandpass filter exclusive isomerization of the first DHP could be observed. The difference spectrum clearly shows the presence of the closed‐open isomer, while for irradiation with 660 nm the open‐open isomer is formed, even at an early stage of the irradiation (Figure 4 b). Due to the low light intensity and the low quantum yield of the first ring‐opening the spectrum of the pure closed‐open isomer could not be obtained experimentally (Figure S26).

**Figure 5:**
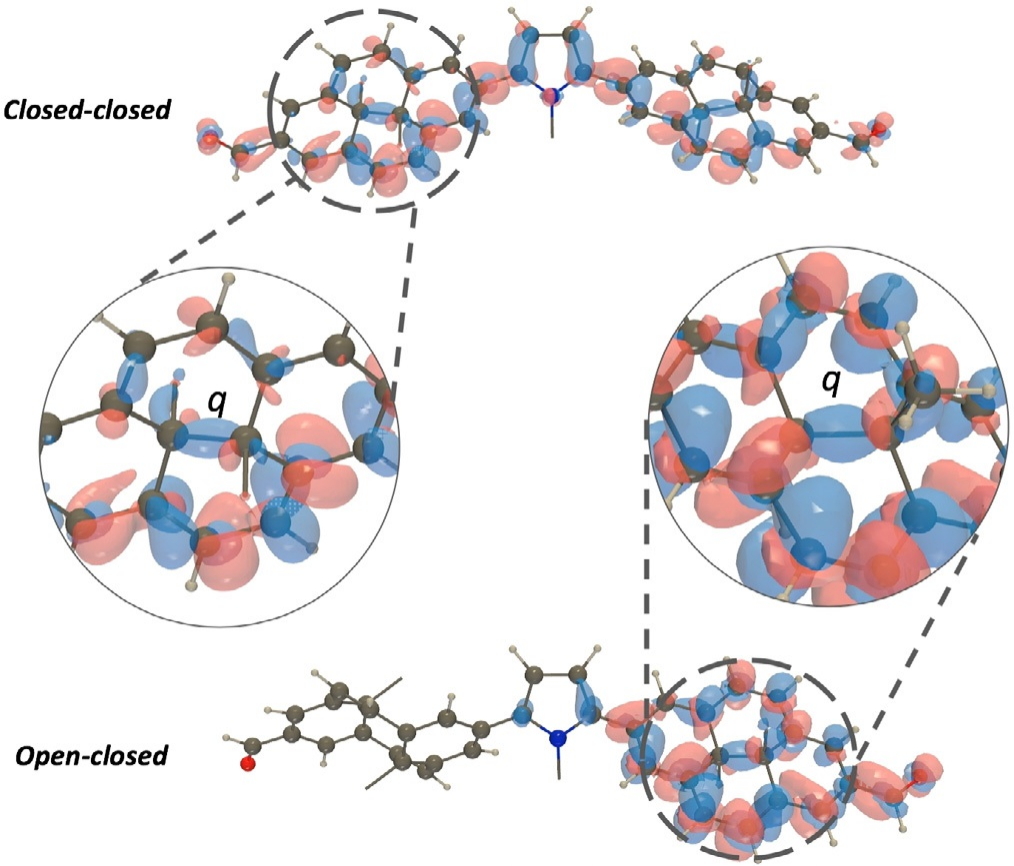
Electron density differences upon excitation to the ring‐ opening state of **PyFm‐dimer** for closed‐closed (top) and closed‐open (bottom). The red and blue lobes indicate regions of increase and decrease of electron density upon excitation. Isosurface value 0.001 au. The alkyl groups were omitted for clarity in the representation.

From a mechanistic standpoint, the general strategy to increase the quantum yield of DHP opening is avoiding the intersection between the two low‐lying excited states of local (LE) and zwitterionic (Z) character to prevent non‐radiative deactivation and pushing the geometry of the biradicaloid CPD precursor as close as possible toward the product CPD geometry.^17^ This strategy was successfully used by Boggio‐Pasqua and Garavelli to optimize DHP derivatives20a and has been applied in this work as well. In the investigated chromophore dimer case, things become more complicated since the interaction between the monomers’ LE and Z states leads to formation of four possible excitons, only one of which exhibits a large oscillator strength. This bright state is a combination of the monomeric Z states with small (8–12 %) contribution of LE character. The coupling between the DHP units stabilizes this ring‐opening state such that it becomes the lowest excited state in the **PyFm**‐**dimer**, thereby avoiding detrimental deactivation via internal conversion to the typically lower lying non‐productive LE state. In contrast, the strong coupling between the DHP moieties implies that the corresponding orbitals are delocalized to a large extent and thus the antibonding character of the transannular bond is less pronounced, disfavoring the ring‐opening process. A simple descriptor of this character is the length of the transannular bond *q* (Figure 5). The computed values of *q* increase in the monomeric DHP from a *S_0_* value of 1.535 Å to 1.609 Å in the optimized geometry of the Z state (Table 1). In the lowest (photoswitching) excited state of the **PyFm**‐**dimer** *q* attains 1.549 Å. Although the excited state population in **PyFm**‐**dimer** cannot be lost into the LE state, the more delocalized nature of the closed‐closed dimer excited state means that the system has to explore a sufficiently large portion of the excited‐state hypersurface to reach the proper CPD precursor geometry. In the open‐closed isomer, the state ordering of the parent molecule is conserved with the *S_1_* state exhibiting Z character, however and in strong contrast, the less delocalized character leads to a significantly longer transannular bond (now 1.579 Å) indicative of a much more effective second ring‐opening process. In that sense, the relocalization of the excited state after the first opening (Figure 5) is responsible for the cooperative effect and the more effective second switching.

**Table 1:** Calculated position of the ring‐opening excited state in the manifold and transannular bond length (*q* in Å) in this excited state. The value of *q* in the ground state is almost uniformly the same (1.535±0.001 Å). Comparison to experimental outcomes is given in the rightmost columns.

	closed‐closed		closed‐open		Experimental Observation	
Molecule	State	*q*	State	*q*	1^st^ Switching	Cooperative
DHP	*S_2_*	1.609	–	–	Yes	–
**PyFm**	*S_1_*	1.549	*S_1_*	1.579	Yes	Yes
**PhCN**	*S_1_*	1.564	*S_2_*	1.569	Yes	Yes
**Ester**	*S_3_*	1.582	*S_2_*	1.587	Yes	n.a.
**CN**	*S_1_*	1.545	*S_1_*	1.568	No	No
**Isoin**	*S_1_* ^[a]^	1.538	*S_2_*	1.550	No	No
**PyCN**	*S_1_*	1.547	*S_1_*	1.566	No	No

[a] For Isoin the *S_1_* state is the one with the largest *f*, but the state with a clear zwitterionic character is *S_3_*.

**Scheme 1:**
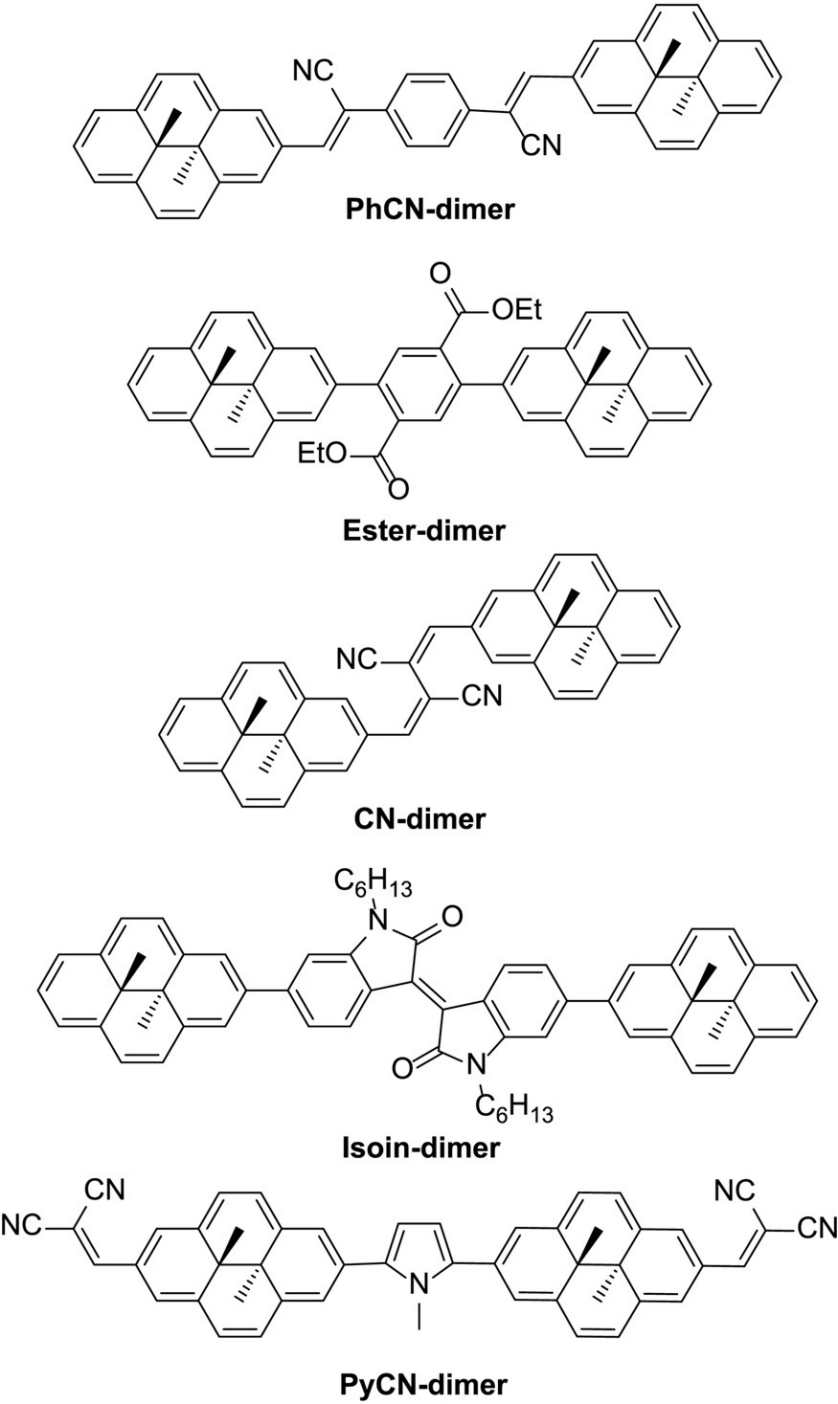
Chemical structures of other investigated DHP dimers.

Due to these promising results for the **PyFm‐dimer** we were interested in examining other dimers as well and exploiting the uncovered cooperative switching phenomenon as a general concept for connecting DHP moieties into longer chains. For this purpose, several DHP dimers were synthesized (Scheme 1) and the experimental findings compared with the predicted switching efficiencies based on our computational approach (see Table 1). The choice of linkers was expanded to acceptors to guarantee the necessary thermal stability to experimentally observe the cooperative effect, i.e., absence or presence of isosbestic points and linearity of absorbance difference diagrams. Comparing our theoretical with our experimental results revealed an interesting interplay of the excited states ordering and transannular bond elongation, required for successful ring opening.

For the **PhCN‐dimer**, the effect of cooperative switching was observed as well. Because of the lack of a strong donor‐acceptor substitution, the difference of the absorption maxima between the isomers is not as pronounced as in the **PyFm‐dimer** and the shift during the thermal back reaction is reduced to 15 nm (Figure S21). The calculations show that the lowest excited state has a large oscillator strength and leads to the first ring‐opening. Relaxation of this excited state yields a geometry with a longer *q* value as compared to the **PyFm‐dimer**. Interestingly, in the intermediate closed‐open isomer the excited state ordering resembles the parent DHP moiety and thus *S_2_* is responsible for photochemical ring‐opening, the bond elongation in this *S_2_* state being large, but less than in the closed‐open **PyFm‐dimer**. The situation differs in the **Ester‐dimer**, in which steric repulsion induces twisting and thus decoupling of the electron‐withdrawing linker. As a result, the photoswitching state is the third one, and one therefore needs to cross two LE surfaces to reach the *Z* minima showing the most elongated *q* value of all tested dimers. Experimentally, ring‐opening of the **Ester‐dimer** was indeed detected but even after 30 min of constant irradiation only a few percent conversion could be achieved (see Figure S22). The thermal back reaction was also quite slow and took about 14 h to be completed; such rate is comparable to the unsubstituted DHP. For the **CN‐dimer**, the extinction in the far‐red region results from the strong conjugation represented by the aligned DHP units (see Figure S23). In contrast to the **Ester‐dimer** the ring‐opening state remains the first of the excited state manifold but its optimization shows only a very small stretching of the transannular bond, in agreement with the lack of any experimentally detectable photoisomerization. For the **Isoin‐dimer** photoisomerization was not observed, which is not surprising due to the combination of factors. The bright excited state is the first in the excited state manifold, but it has no clear ring‐opening character. Indeed, the electron density difference shows that the excited state is mainly localized on the linker, with minimal density depletion on the key CC bond (see Figure S38) and logically only a short *q* is obtained in the minimum. Finally, the **PyCN‐dimer** exhibits a bathochromic shift into the NIR region due to the increased acceptor strength when compared to the **PyFm‐dimer** (see Figure S25). However, even when carrying out irradiation experiments at low temperatures (−60 °C) to prevent the hypothetically fast thermal back reaction, no photoisomerization was observed. This can be rationalized by the weakly stretched transannular bond (*q*=1.547 Å) in the excited state minimum and the large involvement of the accepting moieties in the excitation (see Figure S39). Furthermore, relaxation of the **PyCN‐dimer** yields the smallest gap between the optimized excited state and the ground state (956 nm, <1.3 eV) hinting at fast nonradiative deactivation (and hence no photoswitching). In short, one notices from Table 1 that the three experimentally photoactive dimers are the ones for which the *q* in the relaxed closed‐closed isomer is the largest, whereas the clearest cooperativity effect is observed in the system for which the increase of *q* from the closed‐closed to the closed‐open structure is the largest (i.e., **PyFm‐dimer**).

## Conclusion

In summary, we have successfully designed two DHP dimers that show a cooperative switching behavior, the second photoswitching process being significantly facilitated by the first one. The origin of the cooperativity is the reduced π‐conjugation after the first ring‐opening, increasing the electronic influence of the bridge on the second DHP moiety. For the **PyFm‐dimer** the quantum yield for the second ring‐opening is enhanced by two orders of magnitude. By combining experimental results with computational insights, we could establish a method for evaluating the individual DHP switching efficiencies that will aid in designing new and more sophisticated DHP‐based oligomers and polymers. These should allow to pass the information of a local switching event from neighbor to neighbor, thereby ultimately leading to a directional information transfer cascade along the chain. Moreover, our concept should enable the design of multi‐photochromic systems that can be switched quantitatively and with high efficiencies.

## Conflict of interest

The authors declare no conflict of interest.

## Supporting information

As a service to our authors and readers, this journal provides supporting information supplied by the authors. Such materials are peer reviewed and may be re‐organized for online delivery, but are not copy‐edited or typeset. Technical support issues arising from supporting information (other than missing files) should be addressed to the authors.

Supplementary

## References

[1a] M.-M. Russew , S. Hecht , Adv. Mater. 2010, 22, 3348–3360; 20422653 10.1002/adma.200904102

[1b] A. Goulet-Hanssens , F. Eisenreich , S. Hecht , Adv. Mater. 2020, 32, 1905966. 10.1002/adma.201905966 31975456

[2a] P. Weis , S. Wu , Macromol. Rapid Commun. 2017, 38, 1700220;

[2b] A. B. Kanj , K. Müller , L. Heinke , Macromol. Rapid Commun. 2018, 39, 1700239. 10.1002/marc.201700239 28758288

[3] A. Fihey , A. Perrier , W. R. Browne , D. Jacquemin , Chem. Soc. Rev. 2015, 44, 3719–3759. 25921433 10.1039/c5cs00137d

[4] M. Irie , T. Fukaminato , K. Matsuda , S. Kobatake , Chem. Rev. 2014, 114, 12174–12277. 25514509 10.1021/cr500249p

[5] For a notable exception in a polymeric system, see: F. Stellacci , C. Bertarelli , F. Toscano , M. C. Gallazzi , G. Zotti , G. Zerbi , Adv. Mater. 1999, 11, 292–295.

[6a] T. Kaieda , S. Kobatake , H. Miyasaka , N. Iwai , Y. Nagata , A. Itaya , M. Irie , J. Am. Chem. Soc. 2002, 124, 2015–2024; 11866616 10.1021/ja0115722

[6b] A. Fihey , R. Russo , L. Cupellini , D. Jaquemin , B. Mennucci , Phys. Chem. Chem. Phys. 2017, 19, 2044–2052. 28009859 10.1039/c6cp07458h

[7a] K. Higashiguchi , K. Matsuda , M. Matsuo , T. Yamada , M. Irie , J. Photochem. Photobiol. A 2002, 152, 141–146;

[7b] K. Higashiguchi , K. Matsuda , M. Irie , Angew. Chem. Int. Ed. 2003, 42, 3537–3540; 10.1002/anie.200351751 12900975

[8a] K. Matsuda , M. Irie , J. Am. Chem. Soc. 2001, 123, 9896–9897; 11583554 10.1021/ja0110123

[8b] S. Kobatake , M. Irie , Tetrahedron 2003, 59, 8359–8364.

[9a] K. Higashiguchi , K. Matsuda , N. Tanifuji , M. Irie , J. Am. Chem. Soc. 2005, 127, 8922–8923; 15969548 10.1021/ja051467i

[9b] A. Perrier , F. Maurel , D. Jacquemin , J. Phys. Chem. C 2011, 115, 9193–9203.

[10a] F. Cisnetti , R. Ballardini , A. Credi , M. T. Gandolfi , S. Masiero , F. Negri , S. Pieraccini , G. P. Spada , Chem. Eur. J. 2004, 10, 2011–2021; 15079841 10.1002/chem.200305590

[10b] D. Bléger , J. Dokić , M. V. Peters , L. Grubert , P. Saalfrank , S. Hecht , J. Phys. Chem. B 2011, 115, 9930–9940. 21749103 10.1021/jp2044114

[11a] D. Bléger , Z. Yu , S. Hecht , Chem. Commun. 2011, 47, 12260–12266; 10.1039/c1cc15180k 21998825

[11b] D. Bléger , T. Liebig , R. Thiermann , M. Maskos , J. P. Rabe , S. Hecht , Angew. Chem. Int. Ed. 2011, 50, 12559–12563; 10.1002/anie.201106879 22114009

[11c] C. L. Lee , T. Liebig , S. Hecht , D. Bléger , J. P. Rabe , ACS Nano 2014, 8, 11987–11993; 25345562 10.1021/nn505325w

[11d] C. Weber , T. Liebig , M. Gensler , L. Pithan , S. Bommel , D. Bléger , J. P. Rabe , S. Hecht , S. Kowarik , Macromolecules 2015, 48, 1531–1537.

[12a] V. Boekelheide , J. B. Phillips , J. Am. Chem. Soc. 1967, 89, 1695–1704;

[12b] R. H. Mitchell , V. Boekelheide , J. Am. Chem. Soc. 1974, 96, 1547–1557.

[13a] R. H. Mitchell , Eur. J. Org. Chem. 1999, 2695–2703;

[13b] C. Bohne , R. H. Mitchell , J. Photochem. Photobiol. C 2011, 12, 126–137.

[14a] R. H. Mitchell , T. R. Ward , Y. Wang , P. W. Dibble , J. Am. Chem. Soc. 1999, 121, 2601–2602;

[14b] R. H. Mitchell , S. Bandyopadhyay , Org. Lett. 2004, 6, 1729–1732. 15151400 10.1021/ol049590m

[15] R. H. Mitchell , C. Bohne , Y. Wang , S. Bandyopadhyay , C. B. Wozniak , J. Org. Chem. 2006, 71, 327–336. 16388652 10.1021/jo052153g

[16a] R. H. Mitchell , T. R. Ward , Y. Chen , Y. Wang , S. A. Weerawarna , P. W. Dibble , M. J. Marsella , A. Almutairi , Z.-Q. Wang , J. Am. Chem. Soc. 2003, 125, 2974–2988. 12617665 10.1021/ja0288136

[17] M. Boggio-Pasqua , M. J. Bearpark , M. A. Robb , J. Org. Chem. 2007, 72, 4497–4503. 17506581 10.1021/jo070452v

[18] H.-R. Blattmann , W. Schmidt , Tetrahedron 1970, 26, 5885–5899.

[19] M. A. L. Sheepwash , R. H. Mitchell , C. Bohne , J. Am. Chem. Soc. 2002, 124, 4693–4700. 11971718 10.1021/ja017229e

[20a] M. Boggio-Pasqua , M. Garavelli , J. Phys. Chem. A 2015, 119, 6024–6032; 25582806 10.1021/jp5118773

[20b] D. Roldan , S. Cobo , F. Lafolet , N. Vilà , C. Bochot , C. Bucher , E. Saint-Aman , M. Boggio-Pasqua , M. Garavelli , G. Royal , Chem. Eur. J. 2015, 21, 455–467. 25358895 10.1002/chem.201404858

[21a] K. Ayub , R. Zhang , S. G. Robinson , B. Twamley , R. V. Williams , R. H. Mitchell , J. Org. Chem. 2008, 73, 451–456; 18154301 10.1021/jo7019459

[21b] K. Ayub , R. Li , C. Bohne , R. V. Williams , R. H. Mitchell , J. Am. Chem. Soc. 2011, 133, 4040–4045. 21344935 10.1021/ja1100596

[22a] K. Klaue , Y. Garmshausen , S. Hecht , Angew. Chem. Int. Ed. 2018, 57, 1414–1417; 10.1002/anie.201709554 29243389

[22b] K. Klaue , W. Han , P. Liesfeld , F. Berger , Y. Garmshausen , S. Hecht , J. Am. Chem. Soc. 2020, 142, 11857–11864. 32476422 10.1021/jacs.0c04219

[23] Note that Mitchell and Bandyopadhyay described simultaneous opening of both DHP units in a structurally very different, 4,4′-diethynyl-bridged benzoDHP dimer yet did not investigate the reason for this phenomenon any further. See reference [14b].

[24] H. Mauser , Z. Naturforsch. B 1968, 23, 1025–1030.

[25a] H. Cerfontain , A. Koeberg-Telder , B. H. Bakker , R. H. Mitchell , M. Tashiro , Liebigs Ann. 1997, 873–878;

[25b] A. Bakkar , S. Cobo , F. Lafolet , E. Saint-Aman , G. Royal , J. Mater. Chem. C 2015, 3, 12014–12017;

[25c] S. Cobo , F. Lafolet , E. Saint-Aman , C. Philouze , C. Bucher , S. Silvi , A. Credi , G. Royal , Chem. Commun. 2015, 51, 13886–13889; 10.1039/c5cc04763c 26214006

[25d] M. Boggio-Pasqua , M. L. Vidal , M. Garavelli , J. Photochem. Photobiol. A 2017, 333, 156–164.

[26] M. Kathan , S. Hecht , Chem. Soc. Rev. 2017, 46, 5536–5550. 28857096 10.1039/c7cs00112f

