# Supplementary material for: Highly Cooperative Photoswitching in Dihydropyrene Dimers
Source: Angew Chem Int Ed Engl. 2020 Aug 26;59(43):19352–8. doi: 10.1002/anie.202008523 (PMC7589249; doi:10.1002/anie.202008523)
Supplement: Supplementary file 1 — Supplementary [file ANIE-59-19352-s001.pdf]

Angewandte Chemie
Eine Zeitschrift der Gesellschaft Deutscher Chemiker

Supporting Information

Highly Cooperative Photoswitching in Dihydropyrene Dimers

Pauline Liesfeld, Yves Garmshausen, Simon Budzak, Jonas Becker, André Dallmann, Denis Jacquemin, and Stefan Hecht**

anie_202008523_sm_miscellaneous_information.pdf

Table of Content

1. General Methods S1

2. Synthesis S2

3. UV/vis Absorption Experiments S23

4. NMR Experiments S26

5. Quantum Yield Determination S27

5.1 PyFm-dimer S27

5.2 PyFm-monomer S32

6. Computational Methods S34

7. References S37

1. General Methods

Trans-15,16-dimethyl-15,16-dihydropyrene (DHP),[1] 2-bromo-DHP,[2] *N,N′*-dihexyl-6,6′-bis(4,4,5,5-tetra-methyl-1,3,2-dioxaborolan-2-yl)isoindigo[3] and 2,5-dibromodiethyl-terephtalate[4] were synthesized according to literature procedures. We have recently reported the synthesis of 2-formyl-7-bromo-DHP **1** and 2-formyl-7-[*N*-methyl-2-pyrrole]-DHP **2** (PyFm-monomer).[5] The synthesis of 2-formyl-DHP **3** was also based on a previously reported procedure.[6] All other starting materials and reagents were purchased from TCI, Sigma-Aldrich, Fisher Scientific, and ABCR and used as received. Solvents such as dichloromethane, chloroform, ethanol, ethyl acetate, and petroleum ether were distilled prior to use. In case of reactions under inert atmosphere dry solvents were purchased from Acros Organics. For column chromatography silica gel (Merck 60, 0.040 - 0.063 mm) was used. The corresponding thin-layer chromatography (TLC) was carried out on commercial Merck 60 F_{254} silica gel plates. Visualization was possible solely with the naked eye.

NMR spectra were recorded on a Bruker Avance DPX 300 spectrometer, Bruker Avance 400 spectrometer or Bruker Avance III 500 spectrometer at 25 °C. Residual protonated solvent signals of chloroform and dichloromethane were used as internal standard (^{1}H: δ ($CDCl_3$) = 7.26 ppm, δ (CD_2Cl_2) = 5.32 ppm and ^{13}C: δ ($CDCl_3$) = 77.16 ppm, δ (CD_2Cl_2) = 53.84 ppm). Multiplicity was denoted as follows: singlet (s), doublet (d), triplet (t), quartet (q), quintet/pentet (p) and multiplet (m). NMR-irradiation was performed on a Bruker Avance 600 spectrometer at 15 °C. For mass analysis either a Waters UPLC Acquity with a Waters Alliance System (Waters Separation Module 2695, Waters Diode array Detector 996 and Waters Mass detector ZQ 2000) or Agilent 6210 ESI-TOF (Agilent Technologies, flow rate 4 µl/min, spray voltage 4 kV) was used.

UV/vis spectroscopy was carried out on Agilent Cary 50 and Cary 60 instruments connected to a cryostat from Unisoku Scientific Instruments (temperature accuracy ± 0.1 K) in 10 x 10 mm quartz cuvettes with 3 mL volume. As irradiation sources mounted LEDs from Thorlabs with wavelengths of 530 nm (M530L4, FWHM = 35 nm), 617 nm (M617L3, FWHM = 18 nm), 660 nm (M660L4, FWHM = 20 nm) and 730 nm (M730L5, FWHM = 40 nm) were used in combination with adjustable collimation adapters covering wavelength ranges of 350 – 700 nm (Thorlabs, SM2F32-A) and 650 – 1050 nm (Thorlabs, SM2F32-B). The selective excitation experiment required an additional 730 nm bandpass filter (Thorlabs, FL730-10, FWHM = 10 ± 2 nm). For quantum yield measurements a 500 W mercury lamp coupled with a grating monochromator was employed. Actinometry was performed using 1,2-bis(2,4-dimethyl-5-phenyl-3-thienyl)perfluorocyclopentene[7] in hexane at 22 °C with 579 nm. All spectroscopy experiments were carried out in spectroscopy grade solvents by VWR (Uvasol).

2. Synthesis

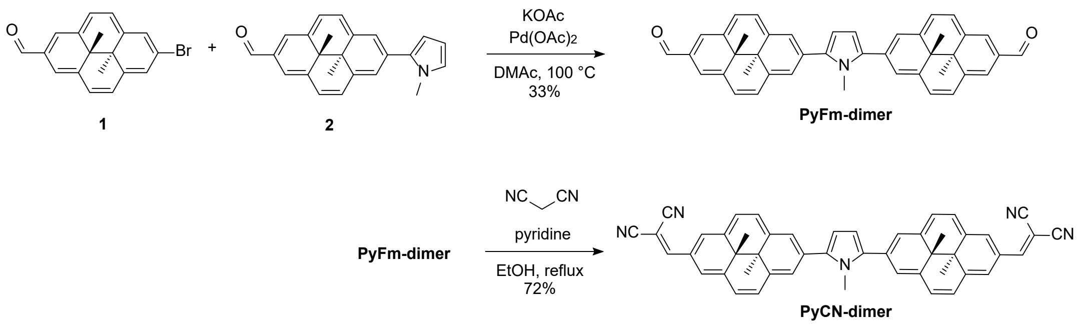

Scheme S1. Synthesis of PyFm-dimer and PyCN-dimer.

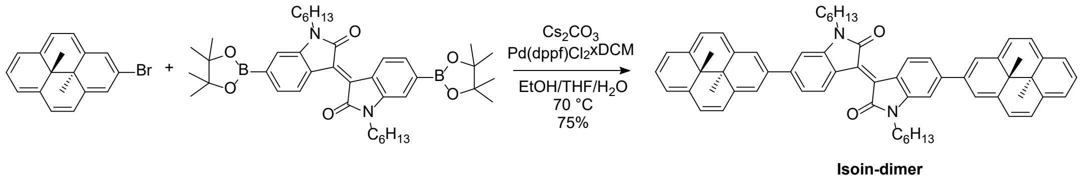

Scheme S2. Synthesis of Isoin-dimer.

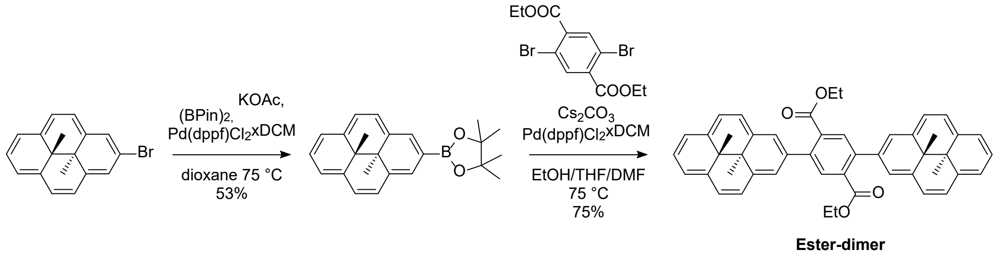

Scheme S3. Synthesis of Ester-dimer.

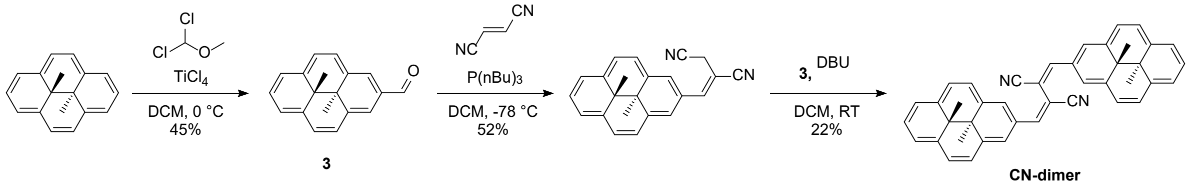

Scheme S4. Synthesis of CN-dimer.

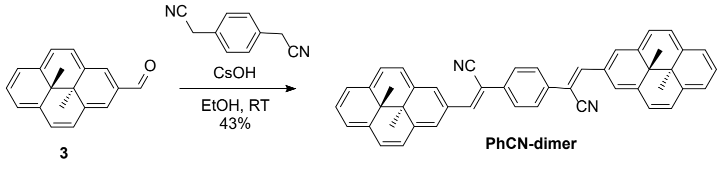

Scheme S5. Synthesis of PhCN-dimer.

PyFm-dimer

A Schlenk tube was charged with 2-formyl-7-[*N*-methylpyrrole]-DHP (31 mg, 0.09 mmol), 2-formyl-7-bromo-DHP (46 mg, 0.14 mmol, 1.5 equiv.), potassium acetate (18 mg, 0.18 mmol, 2.0 equiv.) and 1.0 mL of dry DMAc. The mixture was degassed and put under argon atmosphere three times. Palladium(II) acetate (1 mg, 5 µmol, 5 mol%) was added and the reaction stirred at 100 °C overnight. After allowing the mixture to cool to room temperature, water was added and the mixture was extracted with DCM several times. The combined organic phases were washed with brine and dried over magnesium sulfate. Removal of the solvent under reduced pressure afforded the crude product which was purified by column chromatography (silica, petroleum ether/chloroform = 2:1 to chloroform). The product was obtained as a dark greenish blue solid (18 mg, 0.03 mmol, 33%).

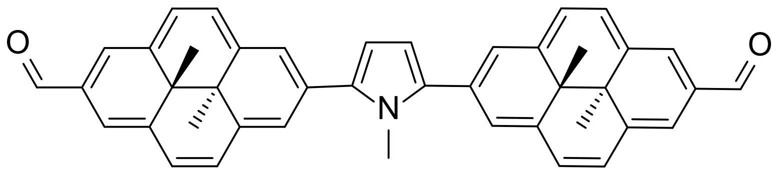

^{1}H-NMR (400 MHz, CDCl$_3$): δ [ppm] = 10.56 (s, 2H), 8.99 (s, 4H), 8.84 – 8.76 (m, 8H), 8.62 (d, *J* = 7.9 Hz, 4H), 7.12 (s, 2H), 4.30 (s, 3H), -3.49 (s, 6H), -3.53 (s, 6H).

^{13}C-NMR (100 MHz, CDCl$_3$): δ [ppm] = 193.4, 141.6, 141.5, 135.6, 130.4, 129.7, 128.9, 125.7, 124.4, 123.6, 114.1, 37.3, 32.1, 31.6, 16.7, 15.2.

HR-MS (ESI$^+$): *m/z* calcd for $C_{43}H_{36}NO_2^+$: 598.275; found: 598.276.

UPLC (20/80 to 95/5 gradient ACN/H$_2$O; basic): t_R = 5.47 min (97% total peak area)

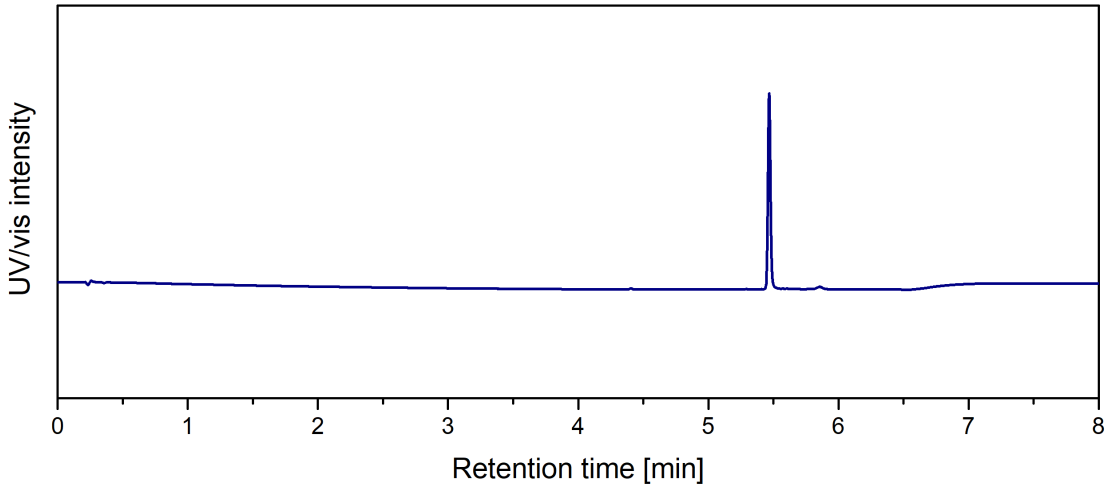

Figure S1. UPLC trace of **PyFm-dimer**.

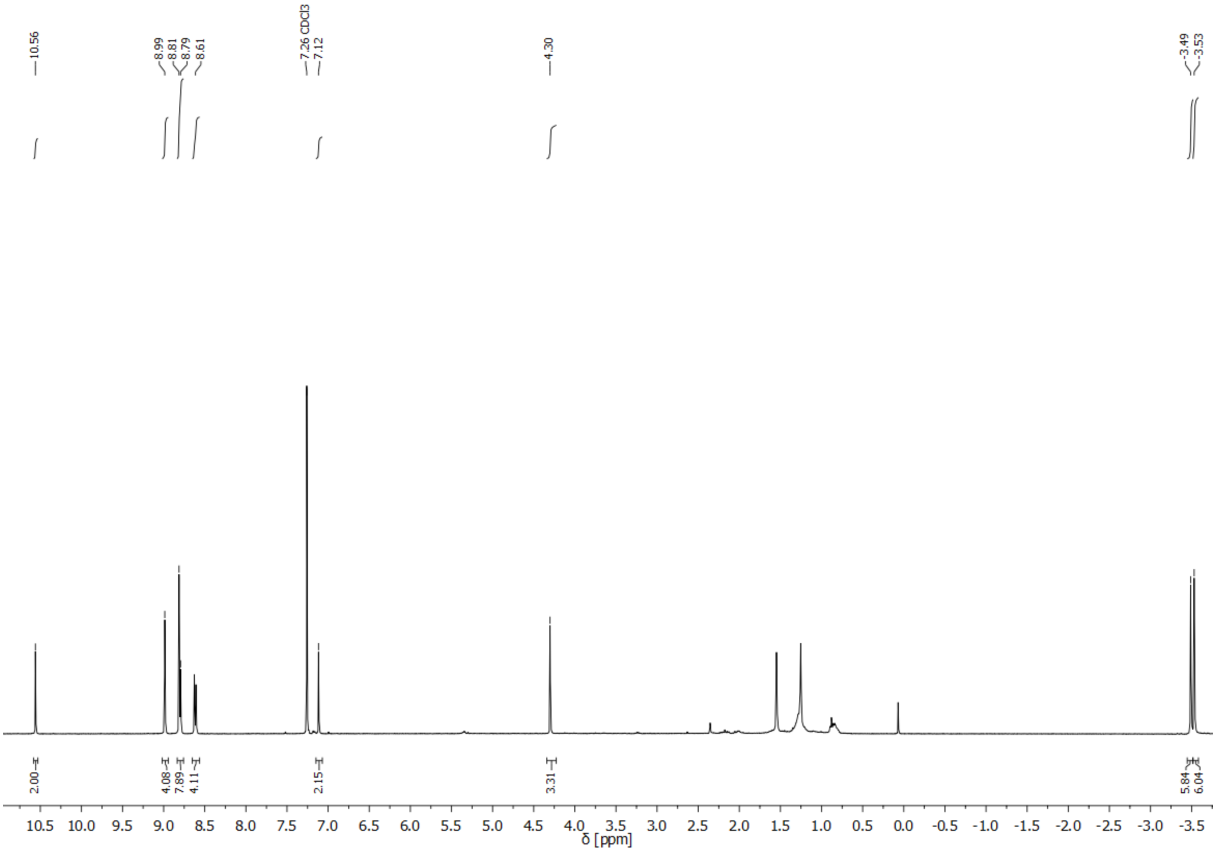

Figure S2. ^{1}H-NMR spectrum of **PyFm-dimer** ($CDCl_3$).

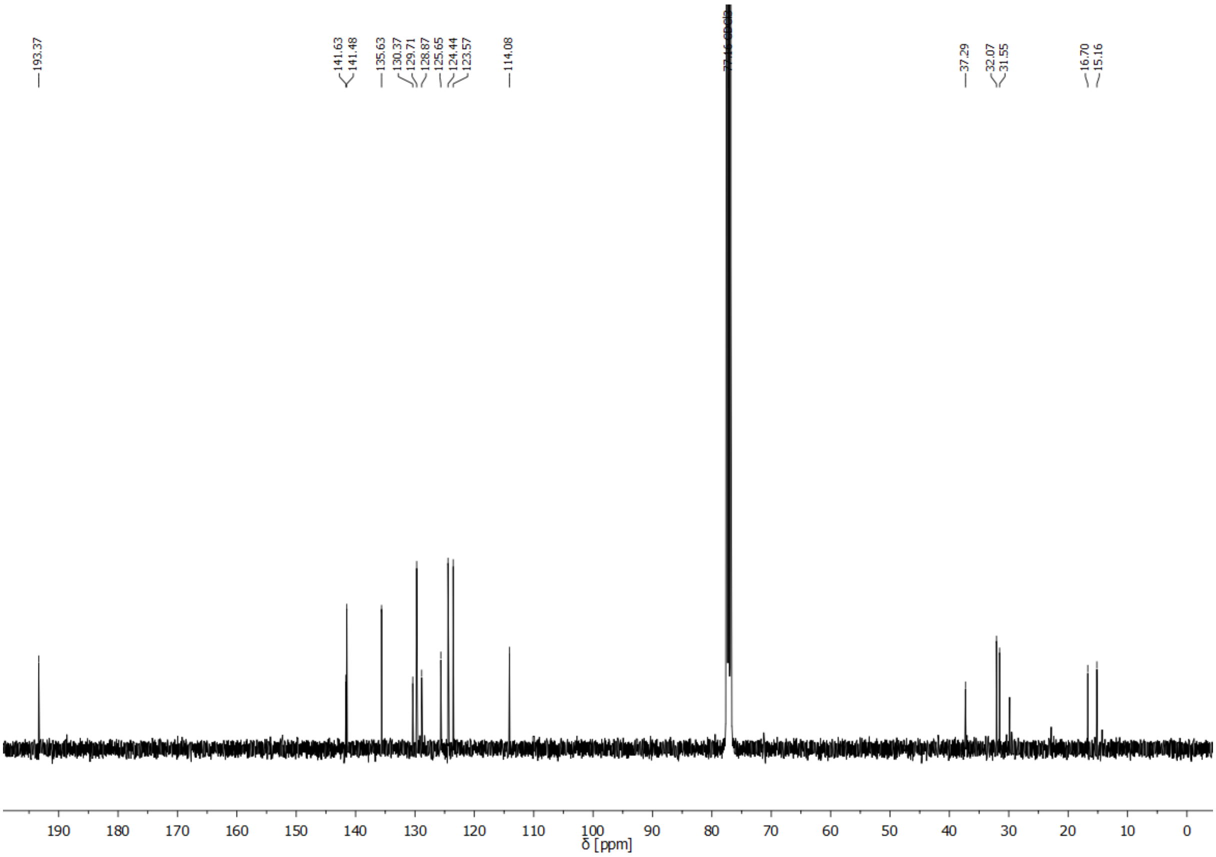

Figure S3. ^{13}C-NMR spectrum of **PyFm-dimer** ($CDCl_3$).

PyCN-dimer

PyFm-dimer (6 mg, 0.01 mmol) was refluxed with malononitrile (2 mg, 0.03 mmol, 3 equiv.) and one drop of pyridine in 2.0 mL of ethanol for 48 h. The solvent was removed under reduced pressure and the residue purified by column chromatography (silica, dichloromethane). The product was obtained as a dark green solid (5 mg, 7 µmol, 72%).

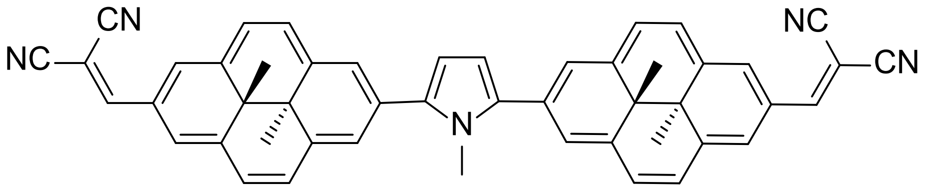

^{1}H-NMR (500 MHz, $CDCl_3$): δ [ppm] = 8.98 (s, 4H), 8.76 (s, 4H), 8.73 (d, *J* = 8.0 Hz, 4H), 8.55 (d, *J* = 8.0 Hz, 4H), 8.13 (s, 2H), 7.18 (s, 2H), 4.32 (s, 3H), -3.22 (s, 6H), -3.25 (s, 6H).

^{13}C-NMR (101 MHz, $CDCl_3$): δ [ppm] = 158.5, 143.7, 142.3, 135.5, 131.8, 131.1, 127.0, 125.2, 124.5, 123.9, 116.1, 115.5, 115.3, 37.2, 32.4, 31.3, 17.9, 15.7.

HR-MS (ESI$^+$): *m/z* calcd for $C_{49}H_{36}N_5^+$: 694.297; found: 694.298.

UPLC (20/80 to 95/5 gradient ACN/H_2O; basic): t_R = 5.60 min (99% total peak area)

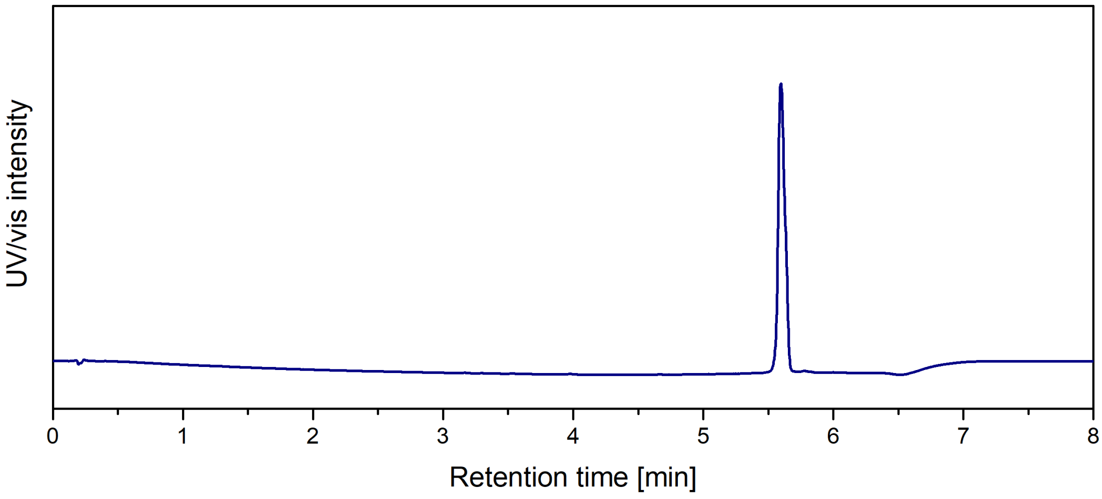

Figure S4. UPLC trace of **PyCN-dimer**.

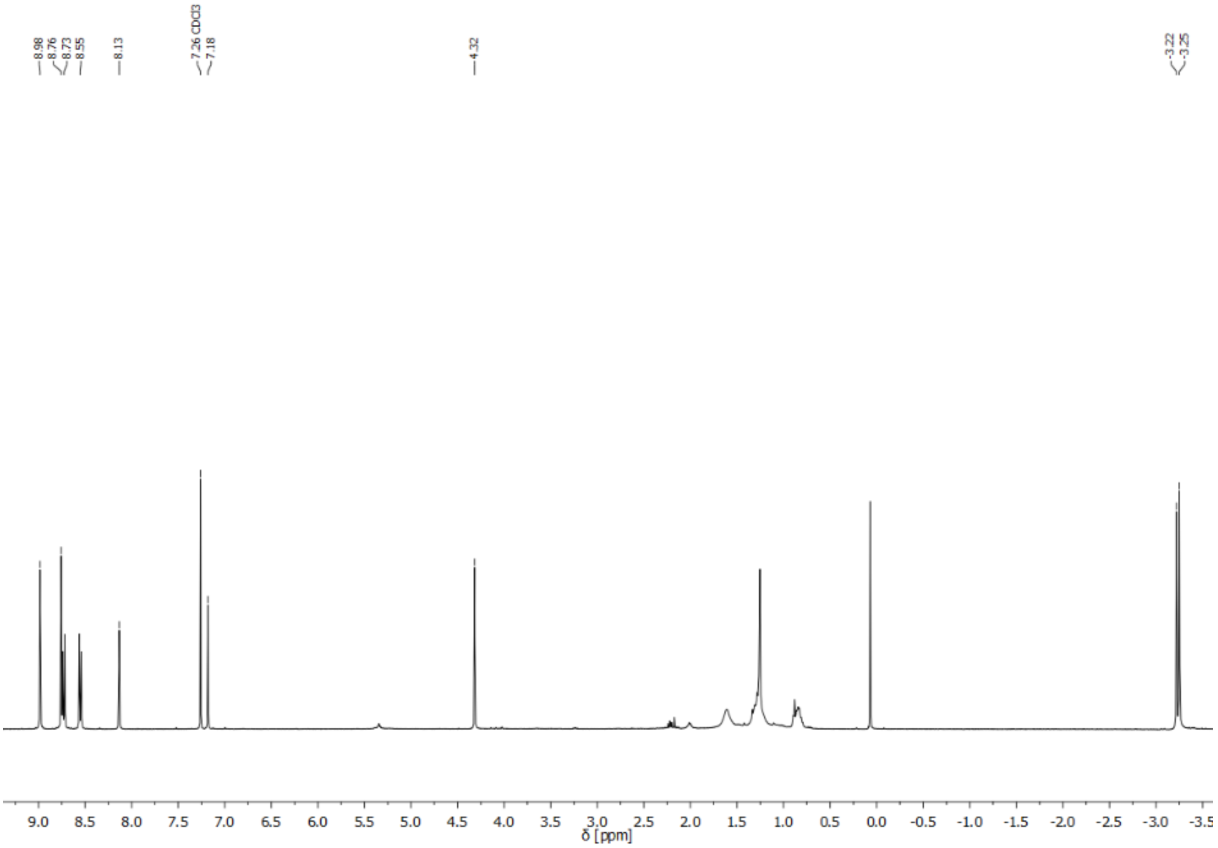

Figure S5. ^{1}H-NMR spectrum of **PyCN-dimer** ($CDCl_3$).

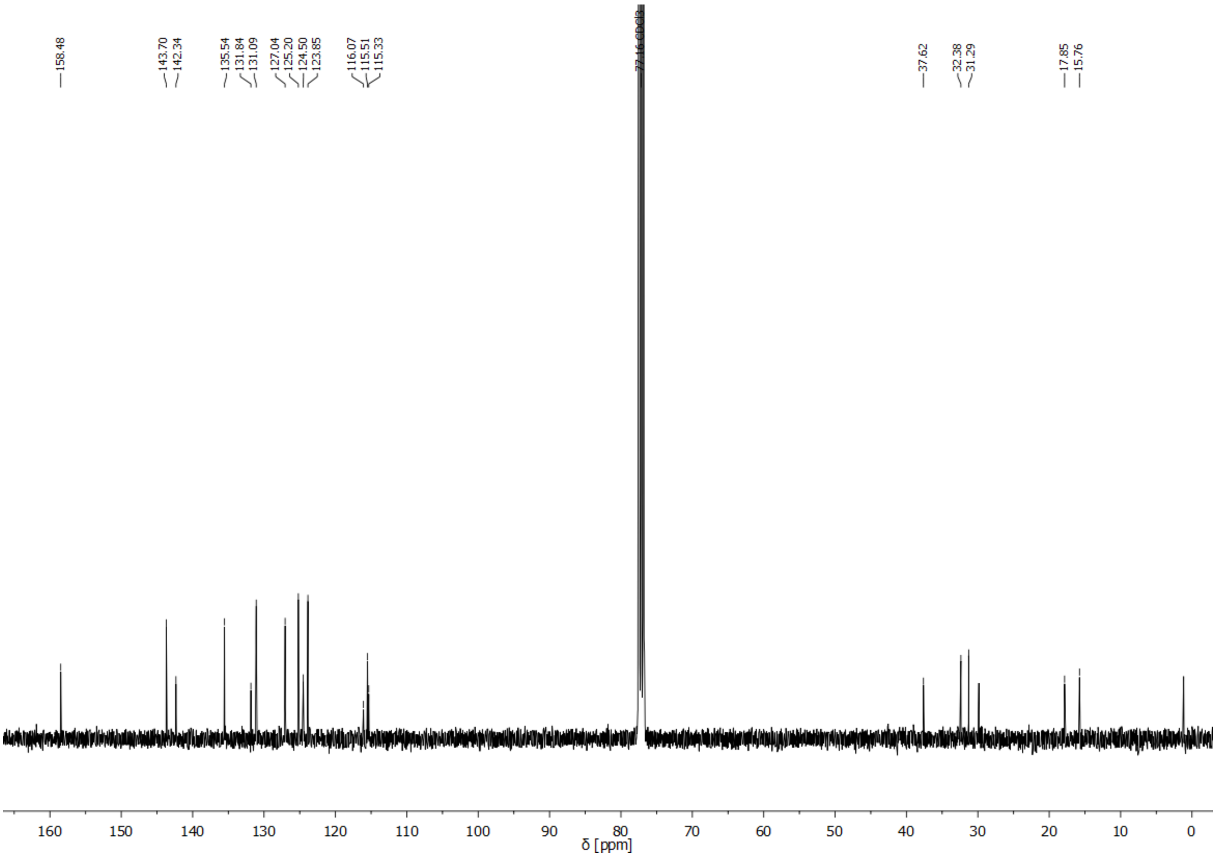

Figure S6. ^{13}C-NMR spectrum of **PyCN-dimer** ($CDCl_3$).

2-(*Trans*-15,16-dimethyl-15,16-dihydropyren-2-yl)-4,4,5,5-tetramethyl-1,3,2-dioxaborolane (DHP boronic acid)

A mixture of 2-bromo-DHP[2,5] (300 mg, 0.96 mmol), bis(pinacolato)diboron (268 mg, 1.06 mmol, 1.1 equiv.) and potassium acetate (283 mg, 2.88 mmol, 3.0 equiv.) was dissolved in 8.0 mL of dry dioxane. The solution was degassed by alternating vacuum and argon several times. Pd(dppf)Cl_2 · CH_2Cl_2 (26 mg, 0.03 mmol, 3 mol%) was added and the mixture was stirred at 75 °C overnight. Water was added and the aqueous phase was extracted with ethyl acetate. The combined organic phases were washed with brine and dried over magnesium sulfate. The solvent was evaporated under reduced pressure and the residue was purified by column chromatography (silica, petroleum ether/methylene chloride = 6:1) to afford the desired product (182 mg, 0.51 mmol, 53%) as a brown solid.

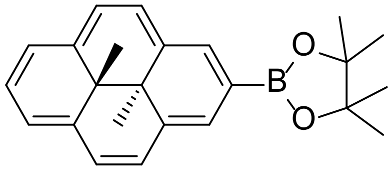

^{1}H-NMR (500 MHz, $CDCl_3$): δ [ppm] = 9.05 (s, 2H), 8.71 (d, *J* = 7.7 Hz, 2H), 8.60 (d, *J* = 7.7 Hz, 2H), 8.56 (d, *J* = 7.7 Hz, 2H), 8.13 (t, *J* = 7.7 Hz, 1H), 1.52 (s, 12H), -4.16 (s, 6H).

^{13}C-NMR (125 MHz, $CDCl_3$): δ [ppm] = 138.7, 135.9, 129.8, 125.8, 124.5, 123.4, 123.2, 84.2, 30.6, 29.7, 25.3, 14.8, 14.3.

HR-MS (ESI$^+$): *m/z* calcd 359.218 for $C_{24}H_{28}BO_2^+$; found: 359.169.

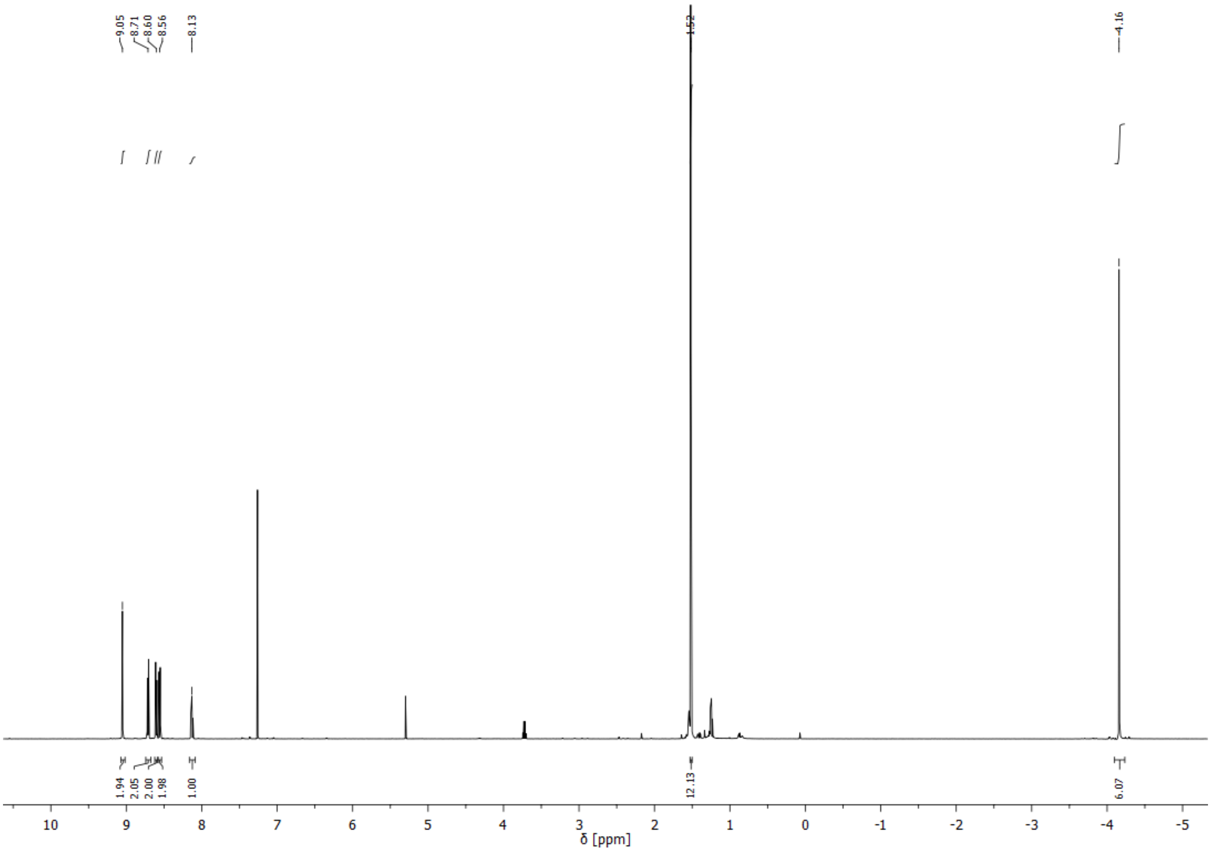

Figure S7. ^{1}H-NMR spectrum of **DHP boronic ester** ($CDCl_3$).

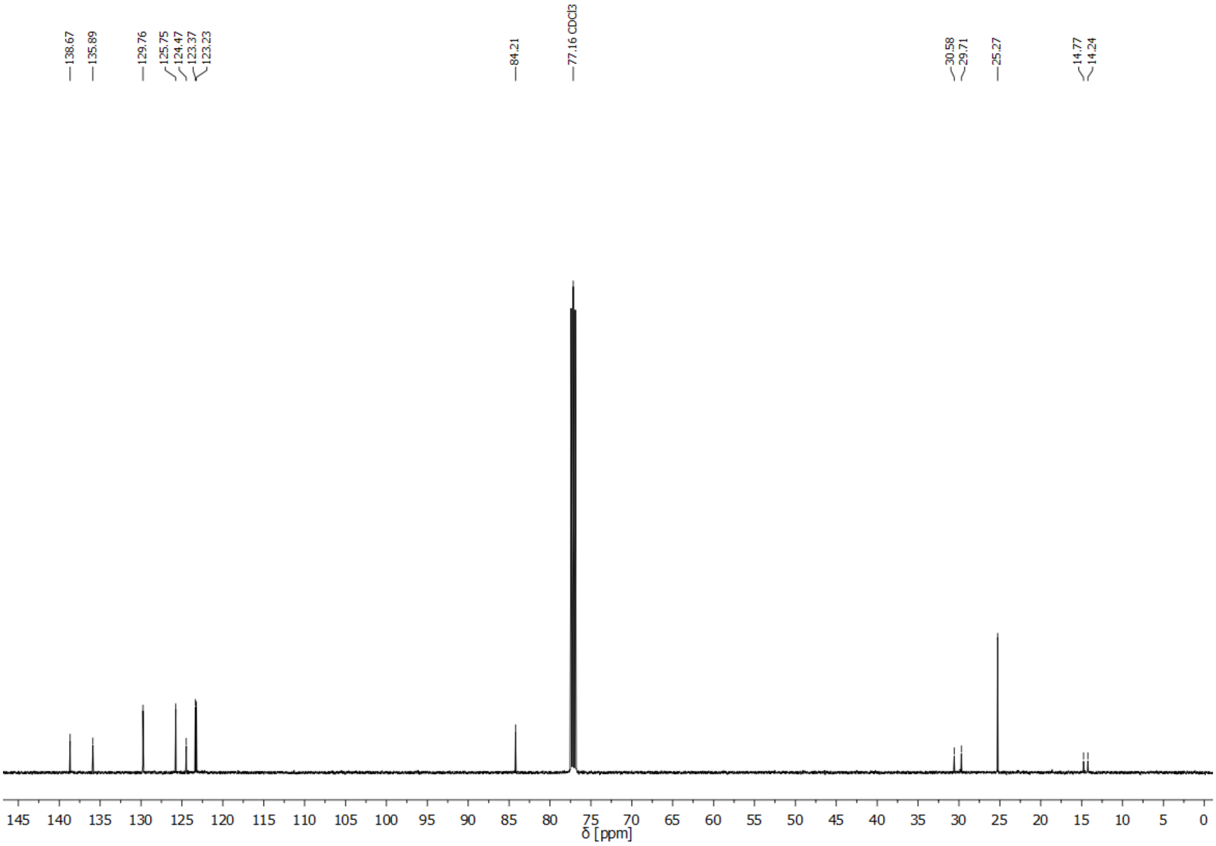

Figure S8. ^{13}C-NMR spectrum of **DHP boronic ester** ($CDCl_3$).

Isoin-dimer

Under argon atmosphere a Schlenk flask was charged with *N,N′*-dihexyl-6,6′-bis(4,4,5,5-tetra-methyl-1,3,2-dioxaborolan-2-yl)isoindigo (48 mg, 0.07 mmol) which was synthesized according to literature procedure.[3] Then, 2-bromo-DHP[2,5] (65 mg, 0.21 mmol, 3 equiv.) and cesium carbonate (98 mg, 0.30 mmol, 4.3 equiv.) were added, followed by 3.0 mL of THF, 1.5 mL of ethanol, and 1.5 mL of water. The solution was degassed and flushed with argon several times. Afterwards Pd(dppf)Cl_2 · CH_2Cl_2 (3 mg, 4 µmol, 5 mol%) was added and the reaction mixture heated at 70 °C overnight. After cooling down, the reaction mixture was diluted with water and extracted with DCM several times. The combined organic phases were washed with brine and dried over magnesium sulfate. The solvent was removed and the residue purified by column chromatography (silica, dichloromethane) which afforded the product as a dark blue solid (24 mg, 0.03 mmol, 38%).

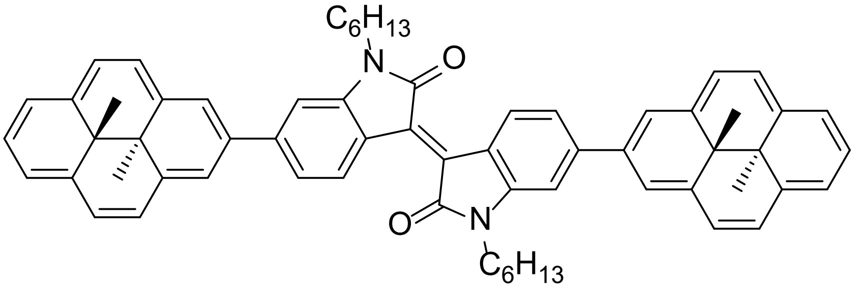

^{1}H-NMR (400 MHz, CD_2Cl_2): δ [ppm] = 9.48 (d, *J* = 8.4 Hz, 2H), 9.05 (s, 4H), 8.77 (d, *J* = 7.7 Hz, 4H), 8.66 (d, *J* = 7.8 Hz, 4H), 8.59 (d, *J* = 7.7 Hz, 4H), 8.11 (t, *J* = 7.7 Hz, 2H), 7.89 (dd, *J* = 8.4, 1.6 Hz, 2H), 7.64 (d, *J* = 1.4 Hz, 2H), 4.04 (t, *J* = 7.2 Hz, 4H), 1.93 (p, *J* = 7.6 Hz, 4H), 1.65 – 1.54 (m, 4H), 1.50 – 1.35 (m, 8H), 0.94 (t, *J* = 7.1 Hz, 6H), -3.90 (s, 6H), -3.94 (s, 6H).

^{13}C-NMR (100 MHz, CD_2Cl_2): δ [ppm] = 169.1, 146.3, 146.3, 138.5, 137.7, 134.1, 132.8, 130.8, 125.5, 124.8, 124.5, 122.6, 122.2, 121.8, 107.6, 40.7, 32.2, 31.3, 30.5, 28.3, 27.4, 23.3, 15.1, 15.1, 14.5.

HR-MS (ESI$^+$): *m/z* calcd for $C_{64}H_{63}N_2O_2^+$: 891.489; found: 891.492.

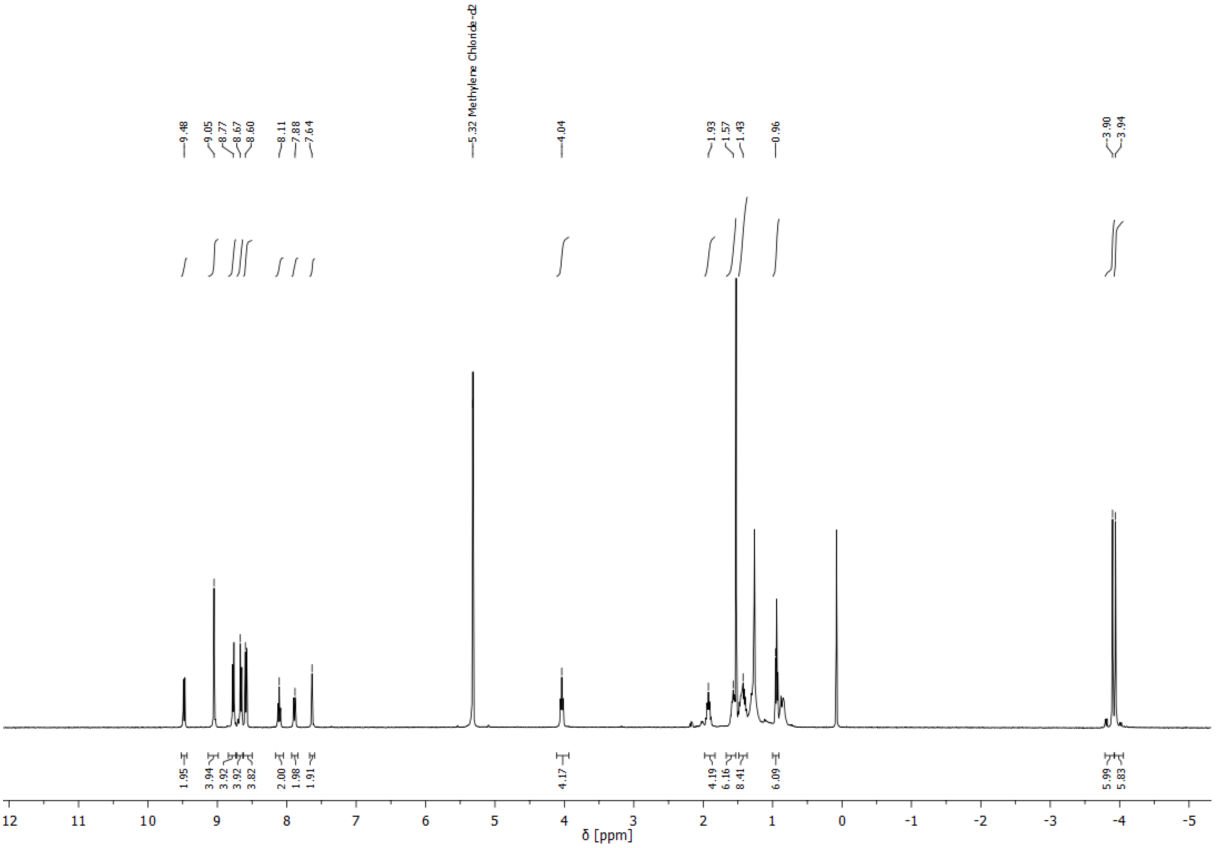

Figure S9. ^{1}H-NMR spectrum of **Isoin-dimer** (CD_2Cl_2).

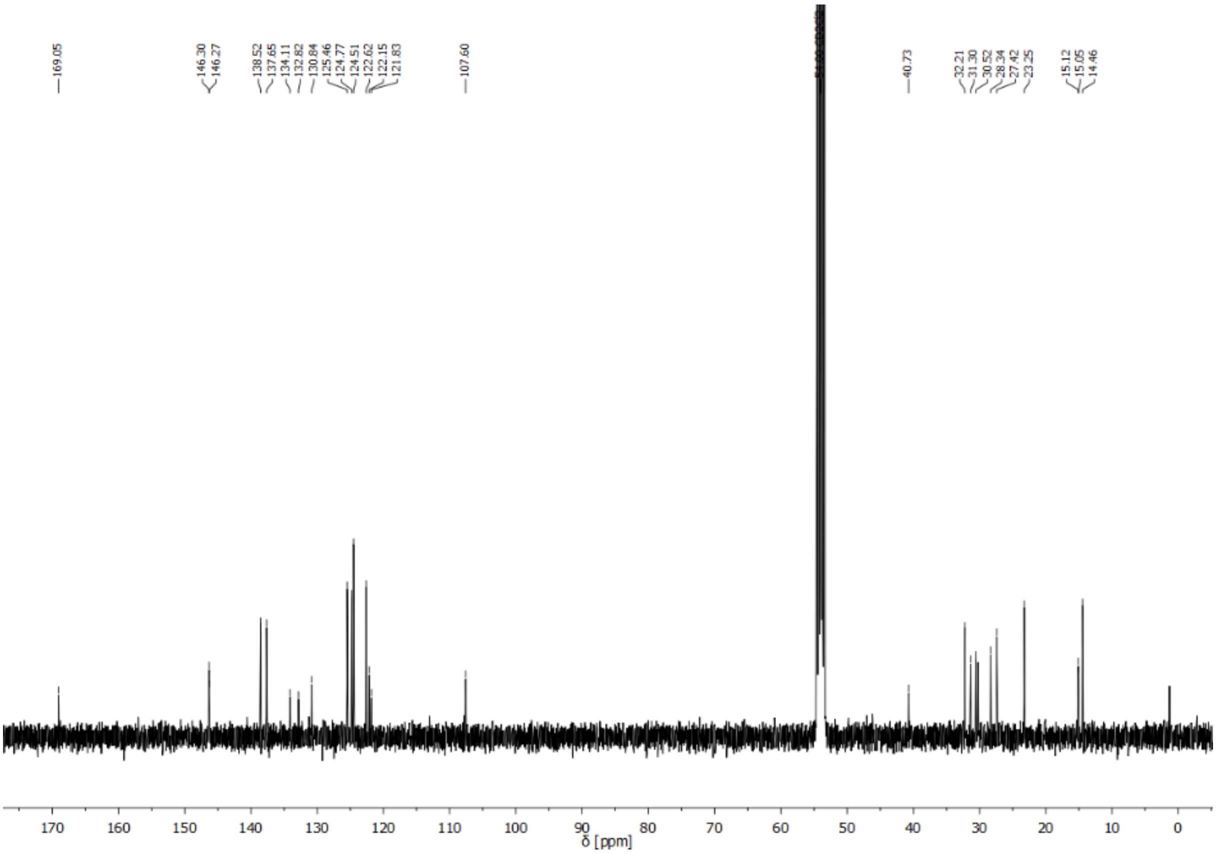

Figure S10. ^{13}C-NMR spectrum of **Isoin-dimer** (CD_2Cl_2).

Ester-dimer

A Schlenk tube was charged with 2,5-dibromodiethylterephthalate (0.077 g, 0.20 mmol) which was synthesized according to literature procedure.[4] Cesium carbonate (0.279 g, 0.86 mmol) and 2-(*trans*-15,16-dimethyl-15,16-dihydropyren-2-yl)-4,4,5,5-tetramethyl-1,3,2-dioxaborolane (0.155 g, 0.42 mmol) were added and everything dissolved in 45.0 mL of a solvent mixture (ethanol/THF/DMF = 4:4:1). The solution was degassed by alternating argon and vacuum three times. Pd(dppf)Cl_2 · CH_2Cl_2 (0.007 g, 0.01 mmol) was added and the reaction mixture was stirred for 3 days at 75 °C. The cooled solution was diluted with water and extracted with ethyl acetate several times. The combined organic phases were washed with brine and dried over magnesium sulfate. The solvent was evaporated under reduced pressure. Purification by column chromatography (silica, petroleum ether/methylene chloride = 1:1) afforded the desired product (0.11 g, 0.15 mmol, 75%) as a red solid.

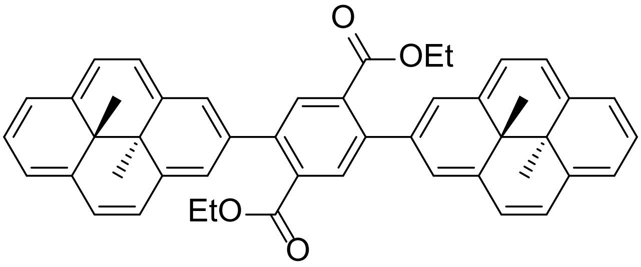

^{1}H-NMR (500 MHz, $CDCl_3$): δ [ppm] = 8.77 (s, 4H), 8.72 (d, *J* = 7.7 Hz, 4H), 8.68 (d, *J* = 7.7 Hz, 4H), 8.62 (d, *J* = 7.7 Hz, 4H), 8.42 (s, 2H), 8.13 (t, *J* = 7.7 Hz, 2H), 4.05 (q, *J* = 7.1 Hz, 4H), 0.89 (t, *J* = 7.1 Hz, 6H), -3.97 (s, 6H), -4.02 (s, 6H).

^{13}C-NMR (125 MHz, $CDCl_3$): δ [ppm] = 169.3, 141.9, 137.5, 136.6, 134.6, 134.0, 133.6, 124.4, 124.2, 124.0, 124.0, 123.7, 61.6, 30.5, 29.8, 14.7, 14.5, 14.0.

HR-MS (ESI$^+$): *m/z* calcd for $C_{48}H_{43}O_4^+$: 683.316; found: 683.318.

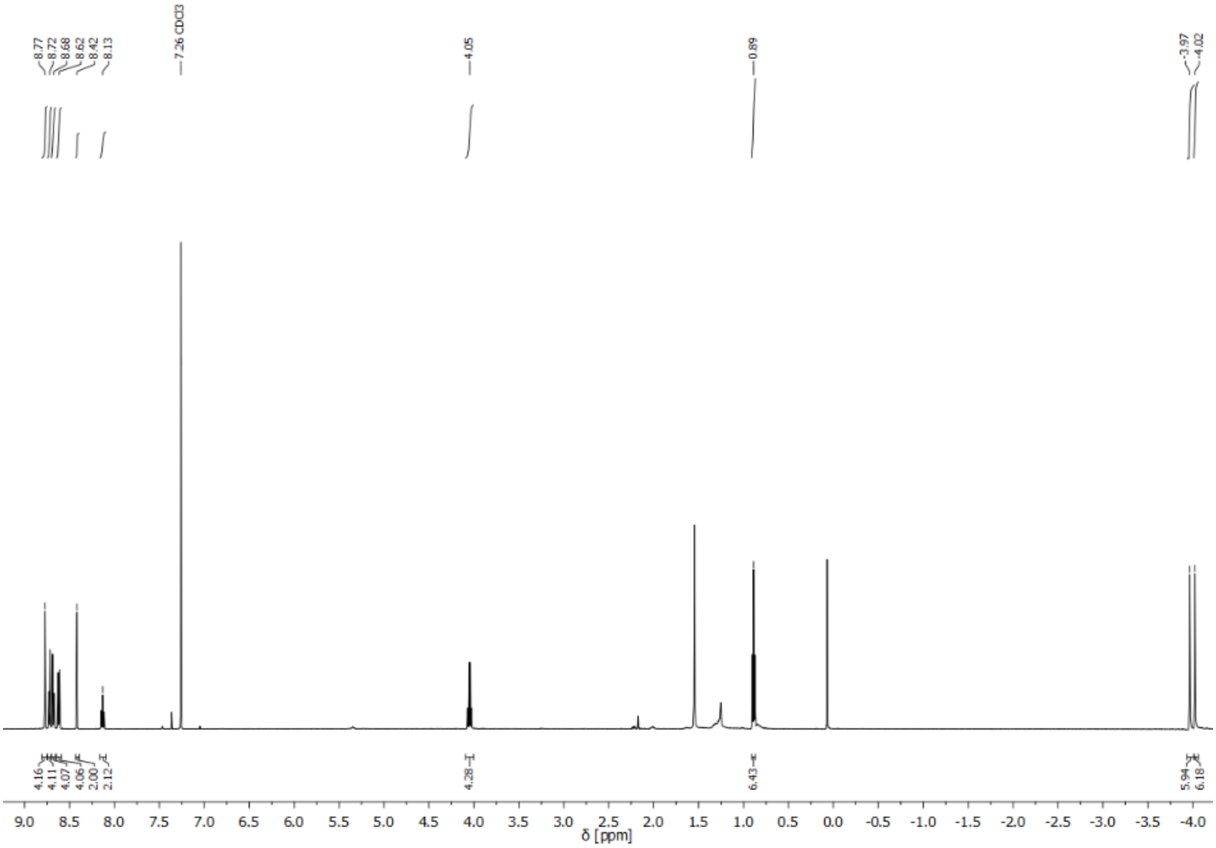

Figure S11. ^{1}H-NMR spectra of **Ester-dimer** ($CDCl_3$).

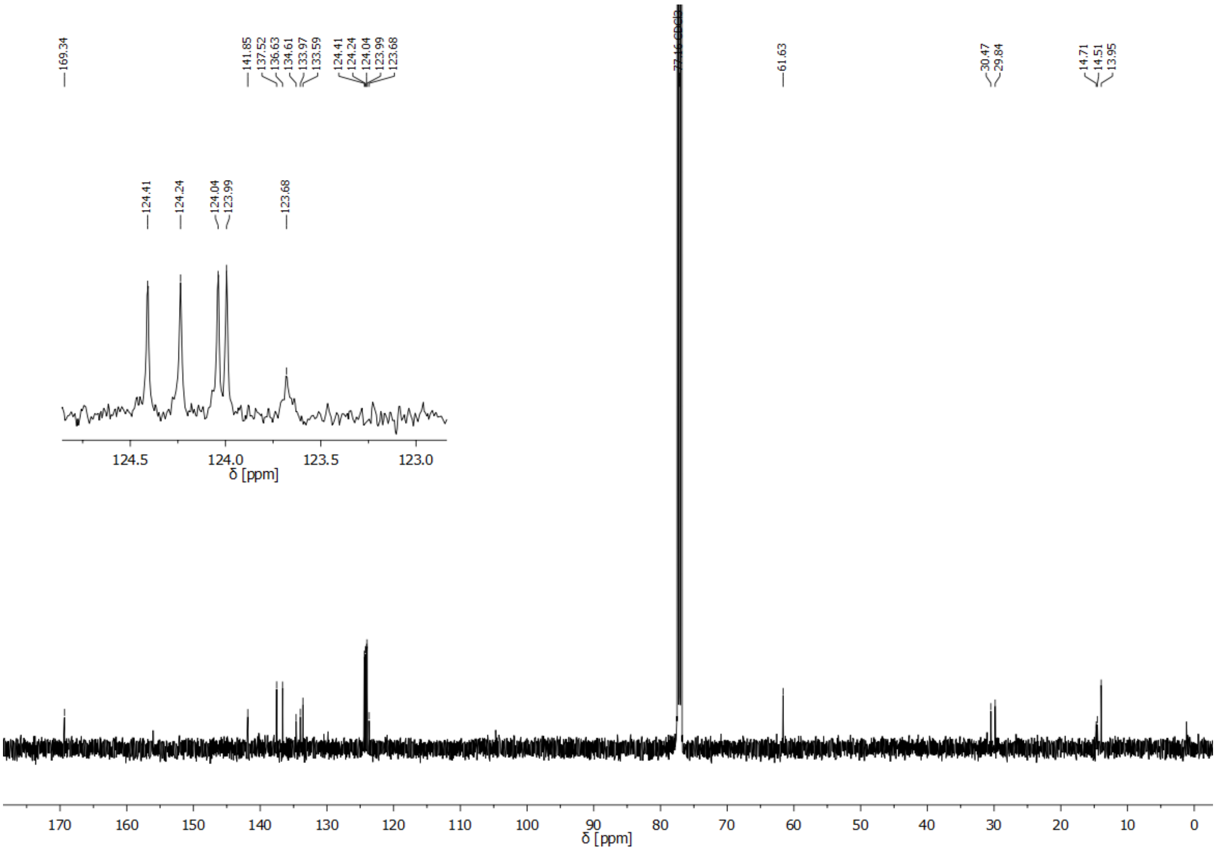

Figure S12. ^{13}C-NMR spectrum of **Ester-dimer** ($CDCl_3$).

2-Formyl-DHP

According to literature procedure[6] DHP (200 mg, 0.86 mmol) was dissolved in 20.0 mL of dry DCM and cooled to 0 °C under argon atmosphere. First, titanium tetrachloride (142 µL, 1.29 mmol, 1.5 equiv.) was added, whereas the solution turned from green to red. Afterwards (dichloromethyl)methylether (101 µl, 1.12 mmol, 1.3 equiv.) was added dropwise, followed by a color change to turquoise. The reaction mixture was stirred at 0 °C for one hour and additional three hours at room temperature. Then the mixture was carefully poured on ice and the aqueous phase extracted with DCM several times. The combined organic phases were washed with brine and dried over magnesium sulfate. Column chromatography (silica, petroleum ether/DCM = 2:1 to DCM) afforded the product as a dark pink solid (100 mg, 0.39 mmol, 45%).

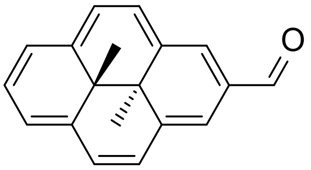

^{1}H-NMR (300 MHz, $CDCl_3$): δ [ppm] = 10.60 (s, 1H), 9.05 (s, 2H), 8.84 (d, *J* = 7.8 Hz, 2H), 8.61 (d, *J* = 7.8 Hz, 2H), 8.56 (d, *J* = 7.8 Hz, 2H), 8.21 (t, *J* = 7.7 Hz, 3H), -3.91 (s, 3H), -3.91 (s, 3H).

^{13}C-NMR (100 MHz, $CDCl_3$): δ [ppm] = 193.7, 141.5, 135.6, 129.0, 127.1, 125.0, 124.3, 124.3, 31.1, 31.3, 15.8, 14.7.

HR-MS (ESI$^+$): *m/z* calcd for $C_{19}H_{17}O^+$: 261.127; found: 261.130.

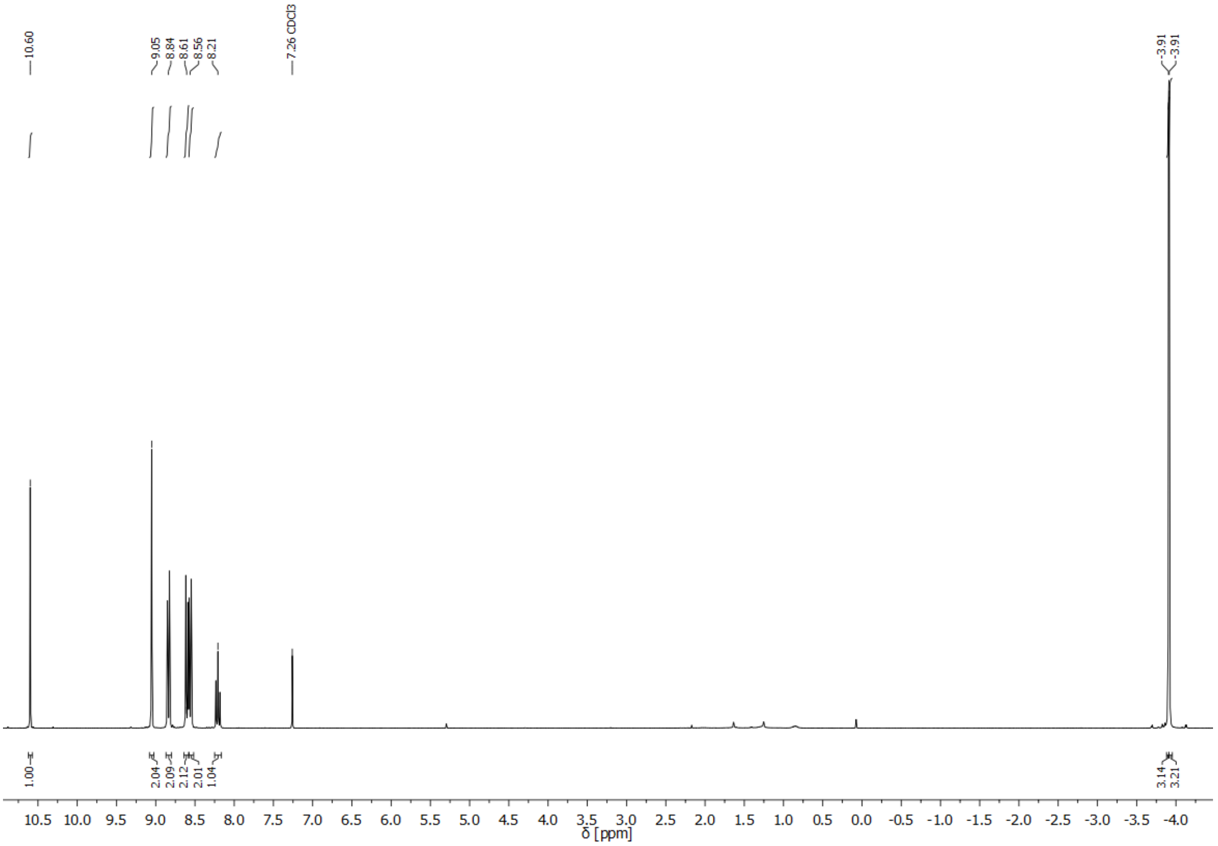

Figure S13. ^{1}H-NMR spectrum of **2-formyl-DHP** ($CDCl_3$).

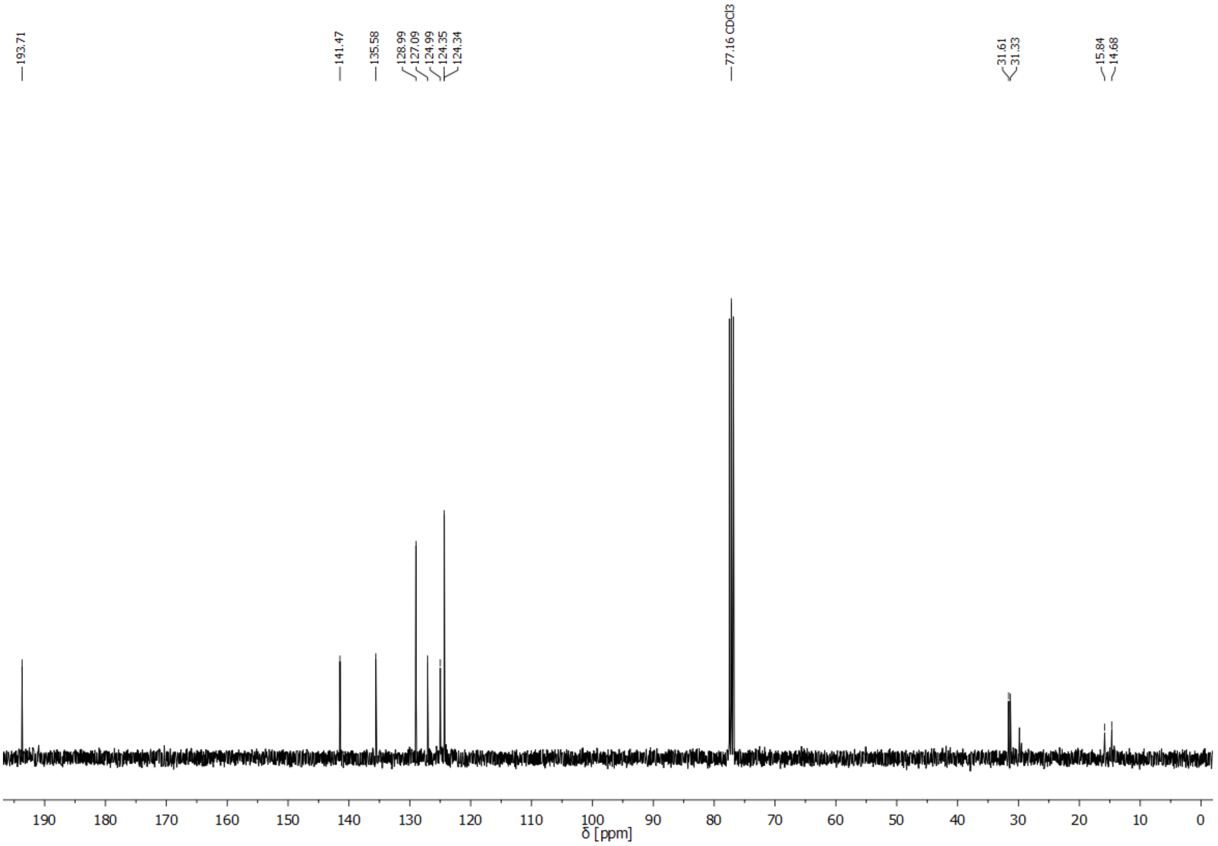

Figure S14. ^{13}C-NMR spectrum of **2-formyl-DHP** ($CDCl_3$).

E-fumaro-nitrile-DHP

In a flame-dried Schlenk tube 2-formyl-DHP (52 mg, 0.20 mmol) and fumaronitrile (17 mg, 0.22 mmol, 1.1 equiv.) were dissolved in 1.5 mL of dry DCM under argon atmosphere. The solution was cooled down to -78 °C and tri-*n*-butylphosphine (59 µl, 24 mmol, 1.2 equiv.) was added dropwise. The reaction mixture was stirred at -78 °C for 30 min and then overnight at room temperature. Water was added and the aqueous phase extracted with ethyl acetate, followed by washing with brine and drying over magnesium sulfate. After the solvent was removed, the product was purified by column chromatography (silica, petroleum ether/ethyl acetate = 2:1) which was obtained as a pink solid (34 mg, 0.11 mg, 52%).

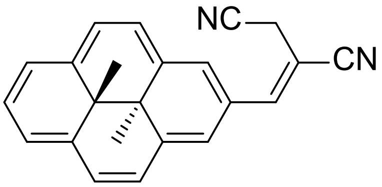

^{1}H-NMR (300 MHz, $CDCl_3$): δ [ppm] = 9.01 (s, 2H), 8.73 (d, *J* = 7.8 Hz, 2H), 8.58 (d, *J* = 7.9 Hz 2H), 8.54 (d, *J* = 7.8 Hz, 2H), 8.15 (t, *J* = 7.7 Hz, 1H), 7.88 (s, 1H), 3.72 (s, 2H), -3.87 (s, 3H), -3.90 (s, 3H).

^{13}C-NMR (75 MHz, $CDCl_3$): δ [ppm] = 148.5, 140.4, 135.8, 127.4, 126.2, 125.5, 124.6, 124.5, 118.5, 115.3, 96.8, 31.3, 30.0, 24.6, 15.6, 14.7.

HR-MS (ESI^{+}): *m/z* calcd for $C_{23}H_{19}N_2^{+}$: 323.154; found: 323.156.

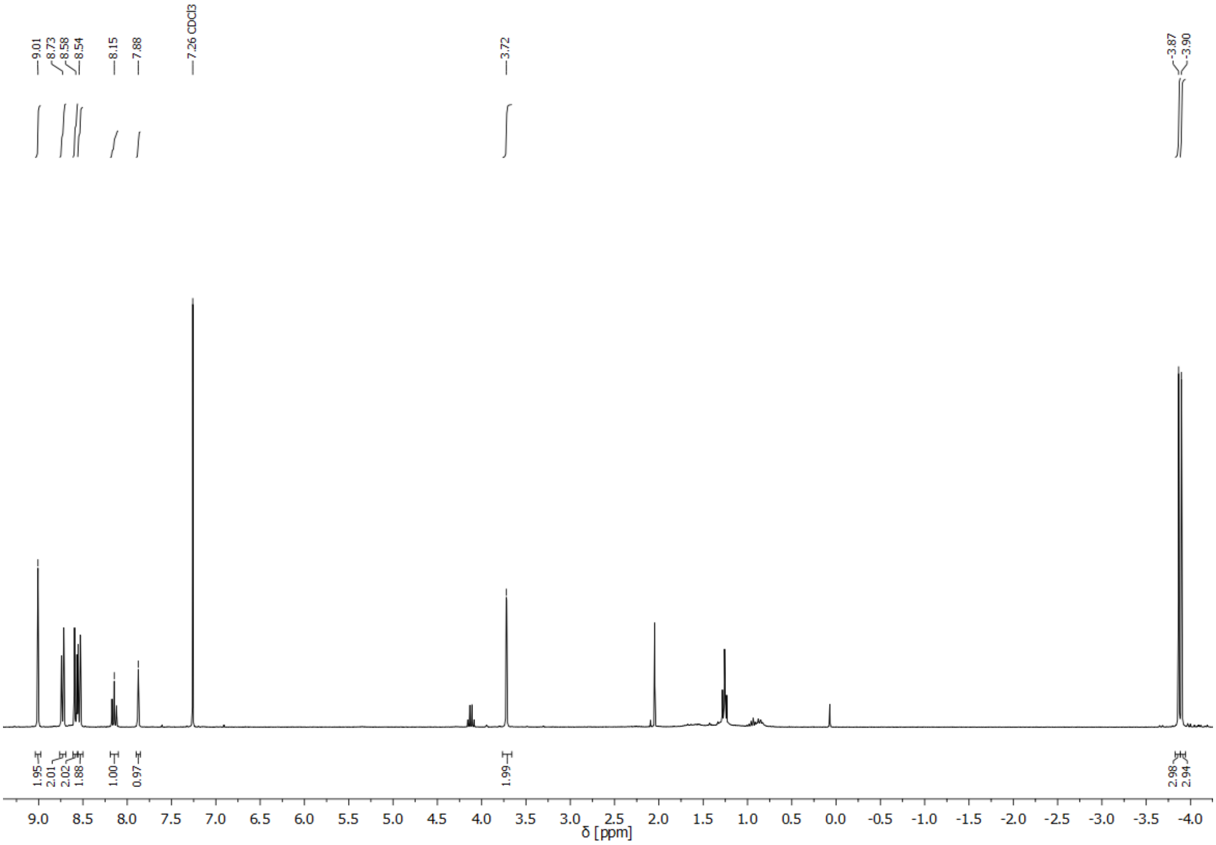

Figure S15. ^{1}H-NMR spectrum of ***E*-fumaronitrile-DHP** ($CDCl_3$).

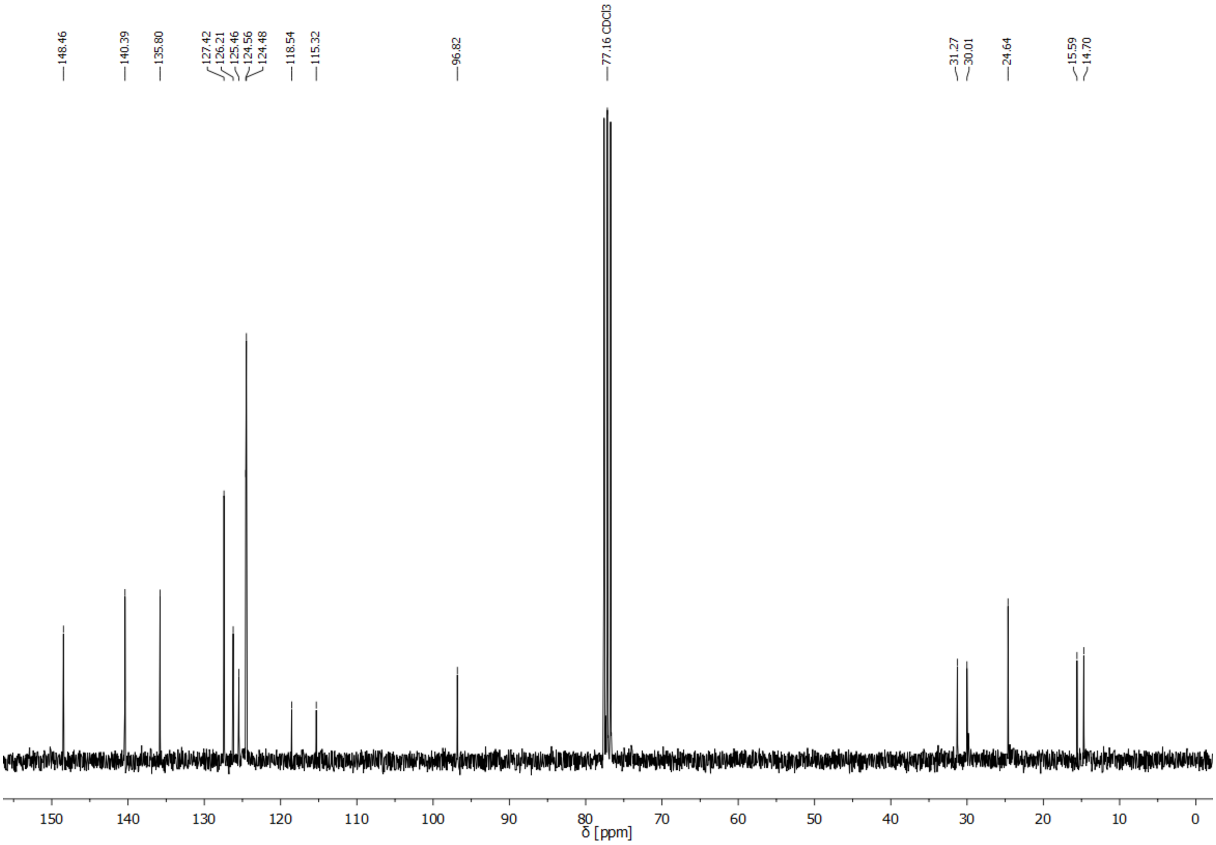

Figure S16. ^{13}C-NMR spectrum of ***E*-fumaronitrile-DHP** ($CDCl_3$).

CN-dimer

In a Schlenk flask 2-formyl-DHP (10 mg, 0.04 mmol) was dissolved in 1.0 mL of dry DCM under argon atmosphere and one drop DBU was added. 2-Fumaronitrile-DHP (13 mg, 0.04 mmol, 1.0 equiv.) dissolved in 2 mL of dry DCM was added dropwise to the aldehyde solution. The reaction mixture was stirred overnight at room temperature. Afterwards the solvent was removed under reduced pressure and the residue purified by column chromatography (silica, petroleum ether/DCM = 1:1 to DCM). Two turquoise bands were isolated corresponding to two different isomers. One of them was identified as the symmetric *E,E*-dimer (5 mg, 9 µmol, 22%).

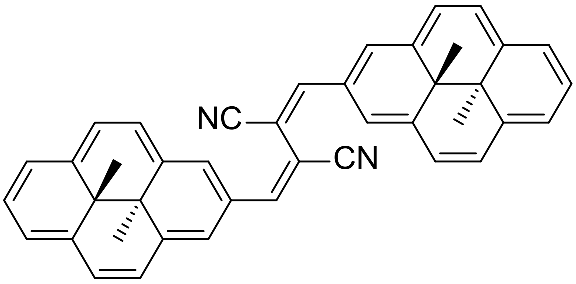

^{1}H-NMR (500 MHz, CD_2Cl_2): δ [ppm] = 9.22 (s, 4H), 8.76 (d, *J* = 7.8 Hz, 4H), 8.59 (d, *J* = 7.8 Hz, 4H), 8.53 (d, *J* = 7.7 Hz, 4H), 8.39 (s, 2H), 8.14 (t, *J* = 7.7 Hz, 2H), -3.65 (s, 6H), -3.70 (s, 6H).

The synthesized amount was not sufficient for ^{13}C-NMR analysis.

HR-MS (ESI$^+$): *m/z* calcd for $C_{42}H_{32}N_2^+$: 564.257; found: 564.258.

UPLC (80/20 to 95/5 gradient ACN/H_2O): t_R = 4.77 min (97% total peak area)

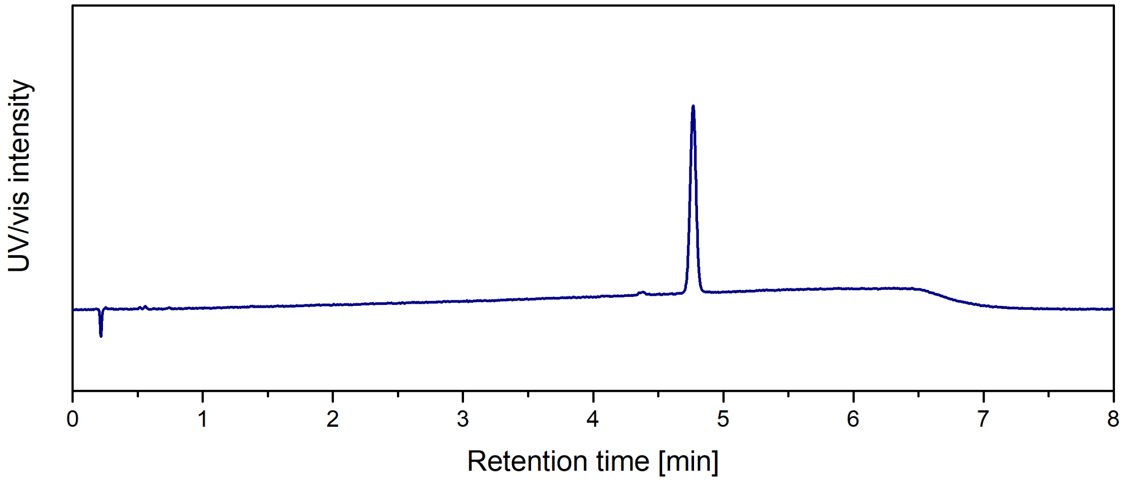

Figure S17. UPLC trace of **CN-dimer**.

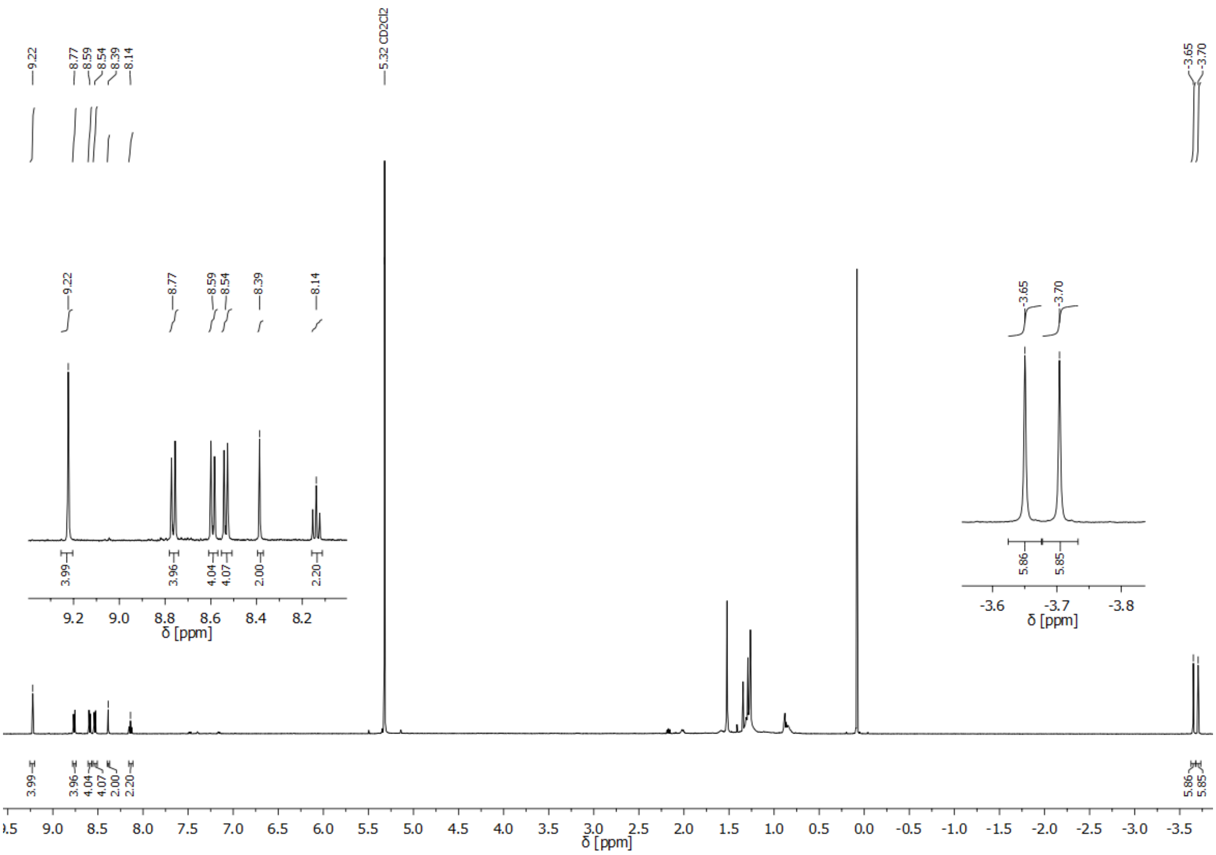

Figure S18. ^{1}H-NMR spectrum of **CN-dimer** (CD_2Cl_2).

PhCN-dimer

Para-xylenedicyanide (17 mg, 0.11 mmol) and 2-formyl-DHP (72 mg, 0.28 mmol, 2.5 equiv.) were suspended in 5.0 mL of ethanol. Afterwards cesium hydroxide (5 mg, 0.03 mmol) was dissolved in 0.5 mL of ethanol and added dropwise. The reaction mixture was stirred overnight at room temperature, whereas the precipitation of the product was observed. After dilution with chloroform it was washed three times with water, followed by brine. The organic phase was dried over magnesium sulfate and the solvent removed under reduced pressure. The residue was purified by column chromatography (silica, chloroform). The obtained crude product was further purified by precipitation into petroleum ether to remove any remaining starting material. The dimer was obtained as a dark blue solid (30 mg, 0.05 mmol, 43 %).

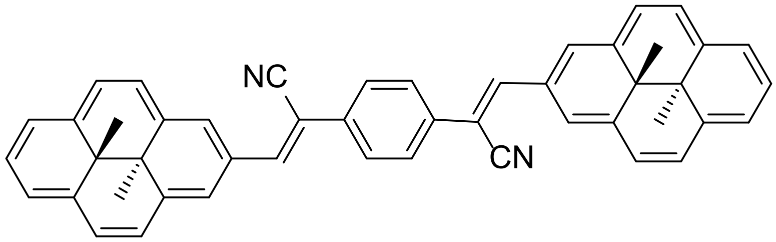

^{1}H-NMR (300 MHz, $CDCl_3$): δ [ppm] = 9.20 (s, 4H), 8.75 (d, *J* = 7.7 Hz, 4H), 8.58 (d, *J* = 7.6 Hz, 4H), 8.53 (d, *J* = 7.8 Hz, 4H), 8.26 (s, 2H), 8.12 (t, *J* = 7.5 Hz, 2H), 8.02 (s, 4H), -3.72 (s, 6H), -3.77 (s, 6H).

Solubility was not sufficient for ^{13}C-NMR analysis.

HR-MS (ESI$^+$): *m/z* calcd for $C_{48}H_{37}N_2^+$: 641.296; found: 641.295.

UPLC (80/20 to 95/5 gradient ACN/H_2O): t_R = 4.79 min (94% total peak area)

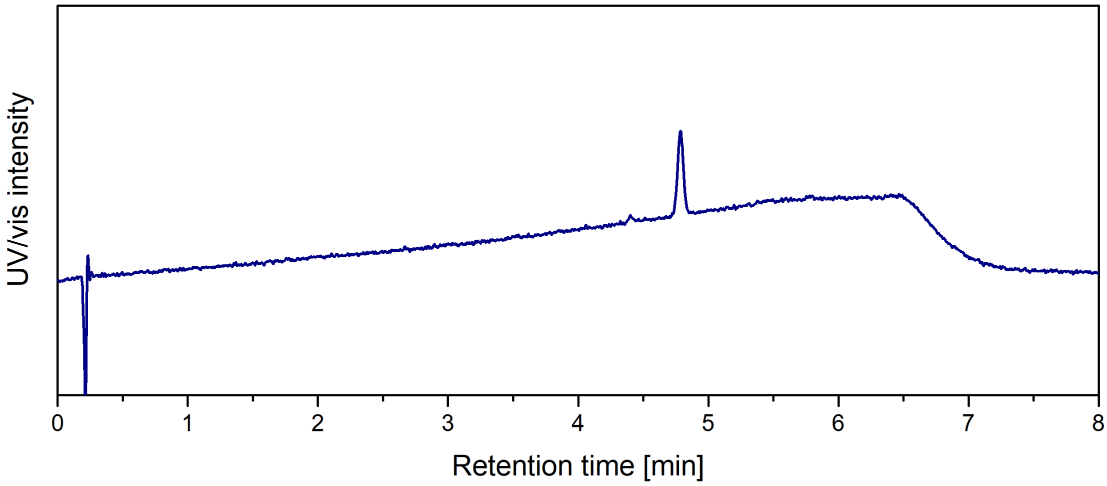

Figure S19. UPLC trace of **PhCN-dimer**.

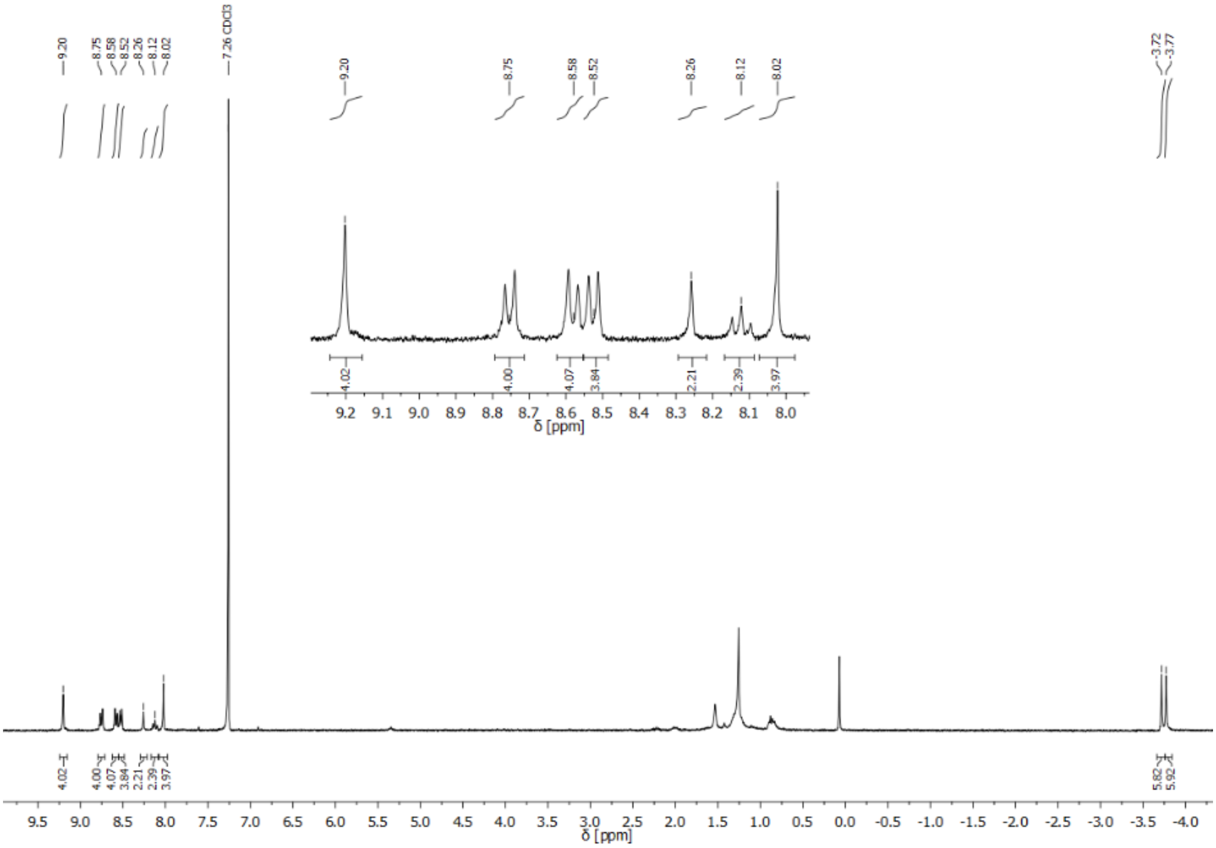

Figure S20. ^{1}H-NMR spectrum of the **PhCN-dimer** ($CDCl_3$).

3. UV/vis Absorption Experiments

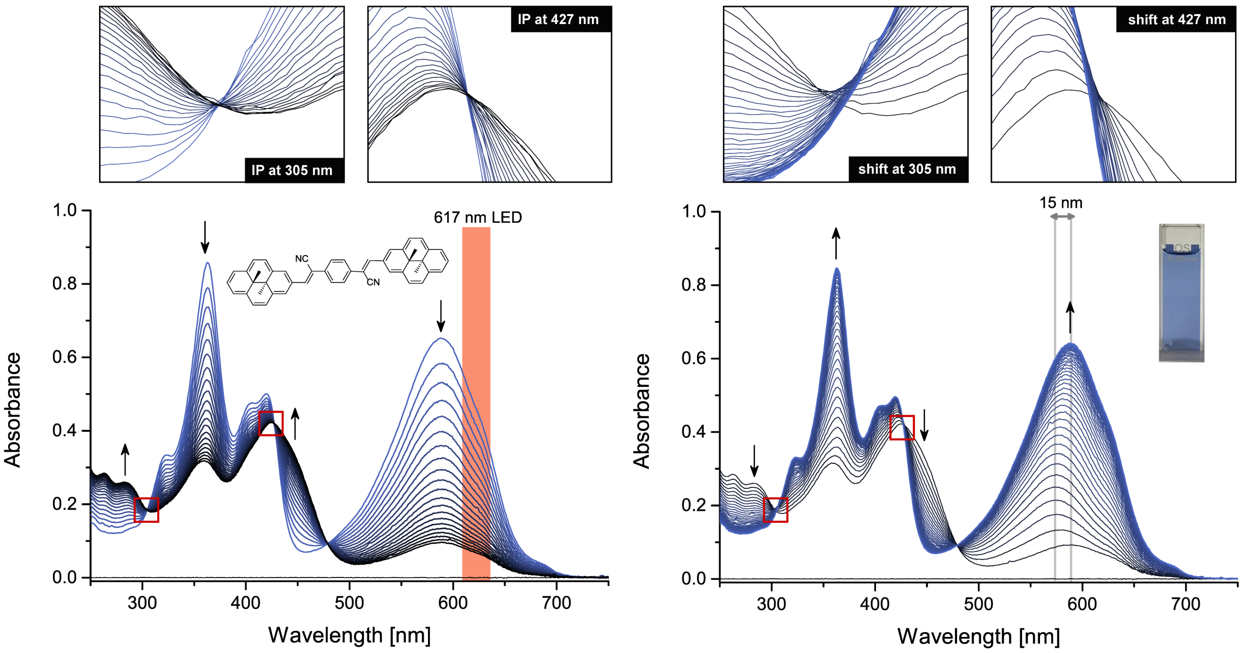

Figure S21. UV/vis spectral changes upon irradiation with a 617 nm LED (left, 2 min between consecutive spectra) and during thermal back reaction (right, 2 min between consecutive spectra) of **PhCN-dimer** at 10 °C in DCM ($1.8 \cdot 10^{-5}$ M). Inset above show the presence or absence of isosbestic points in enlarged spectral regions.

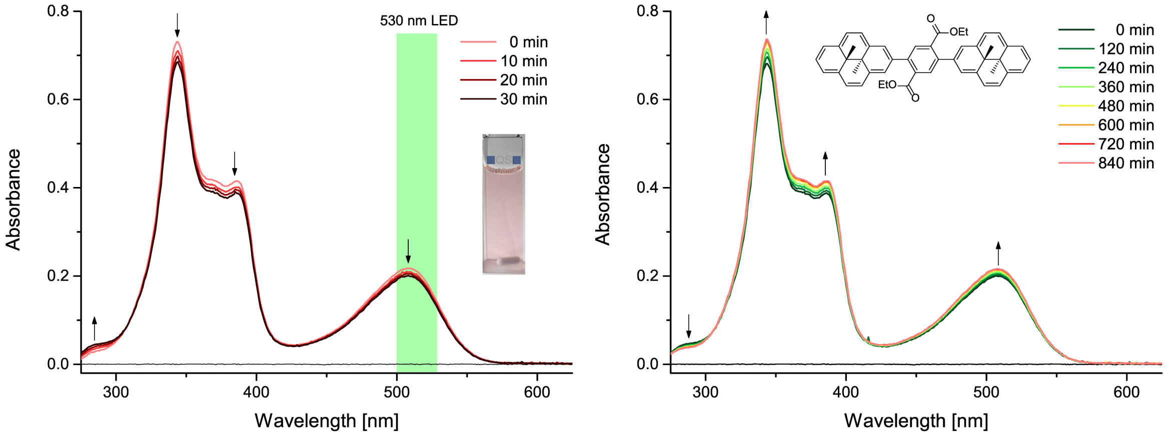

Figure S22. Irradiation with 530 nm LED (left) and thermal back reaction (right) of **Ester-dimer** ($6.16 \cdot 10^{-6}$ M) at 20 °C in degassed THF.

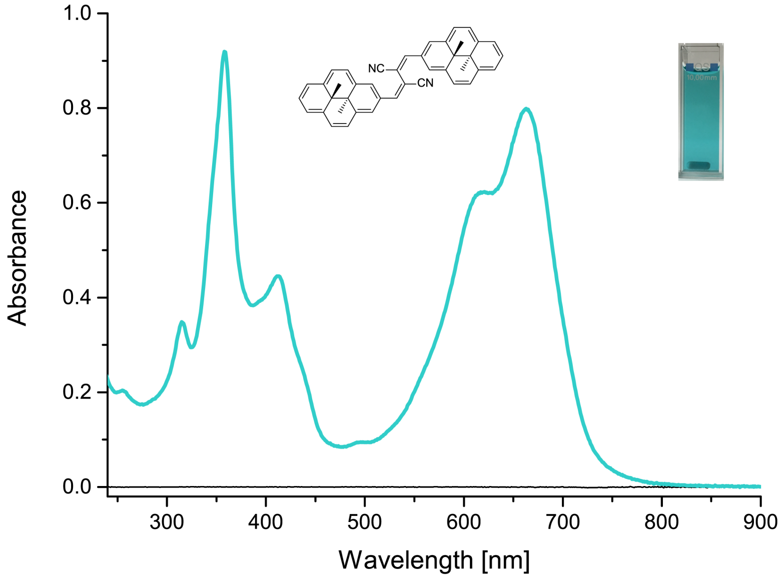

Figure S23. UV-vis absorption spectrum of **CN-dimer** in DCM at 20 °C.

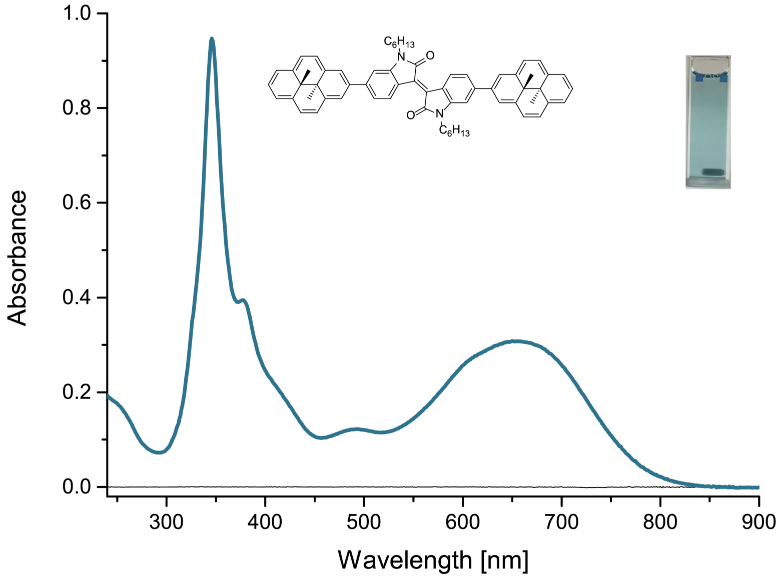

Figure S24. UV-vis absorption spectrum of **Isoin-dimer** ($2.16 \cdot 10^{-5}$ M) in DCM at 20 °C.

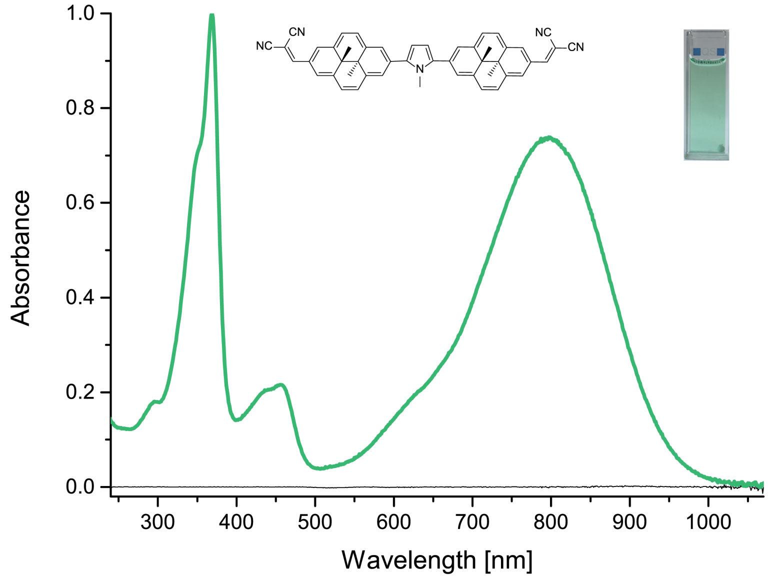

Figure S25. UV-vis absorption spectrum of **PyCN-dimer** (1.25 · 10^{-5} M) in DCM at 20 °C.

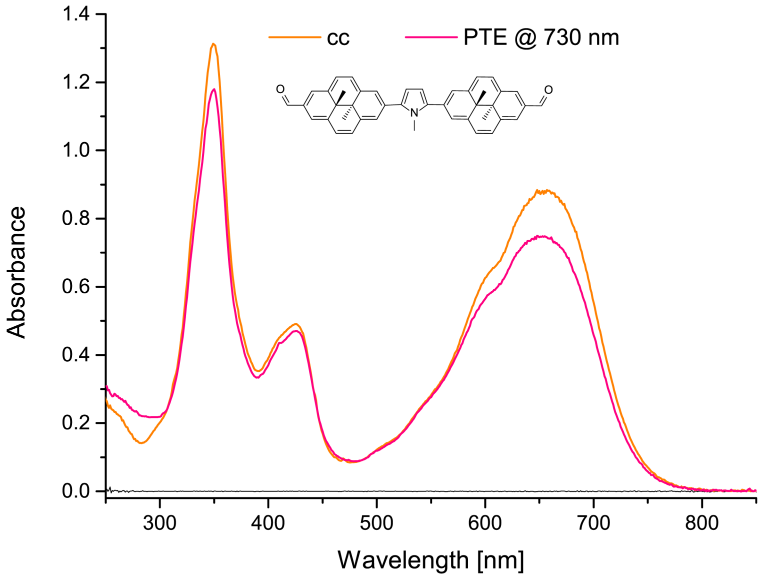

Figure S26. Initial spectrum of **PyFm-dimer** and spectrum of photothermal equilibrium after irradiation with 730 nm at 0 °C in THF (1.31 · 10^{-5} M).

4. NMR Experiments

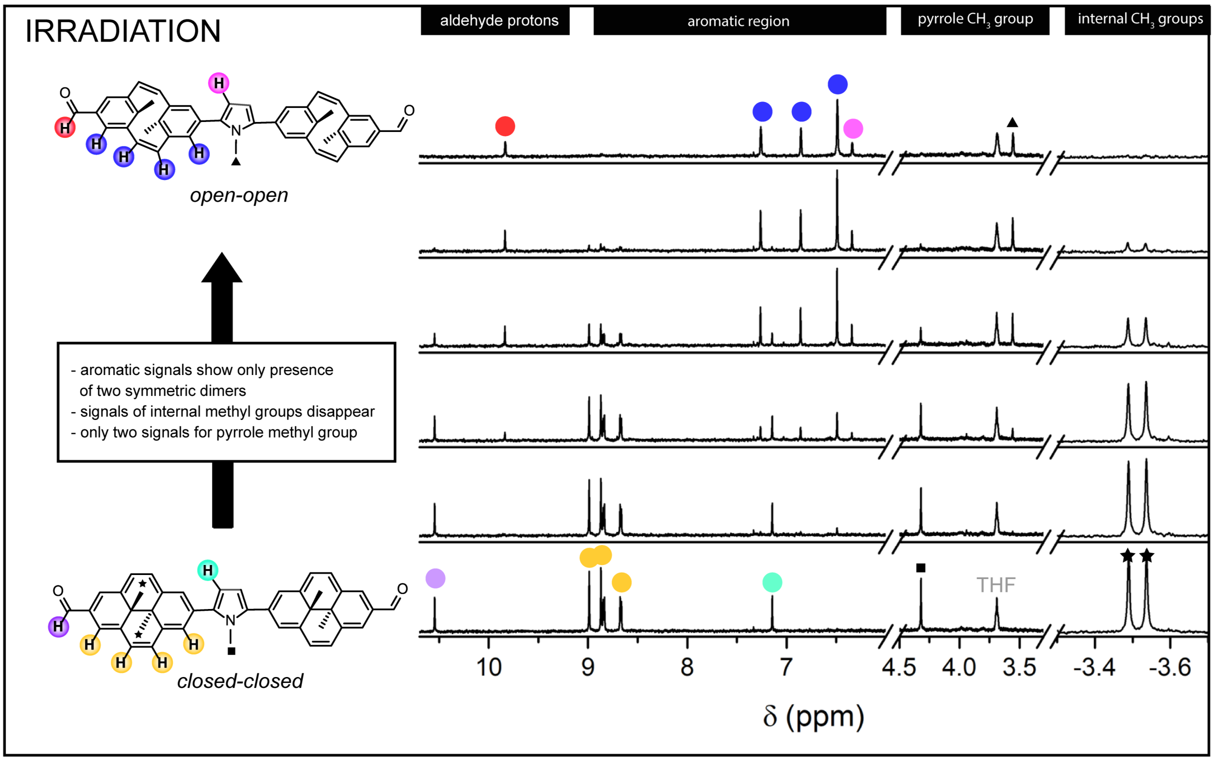

Figure S27. NMR-spectra of **PyFm-dimer** (CD_2Cl_2) during 660 nm irradiation at 15 °C.

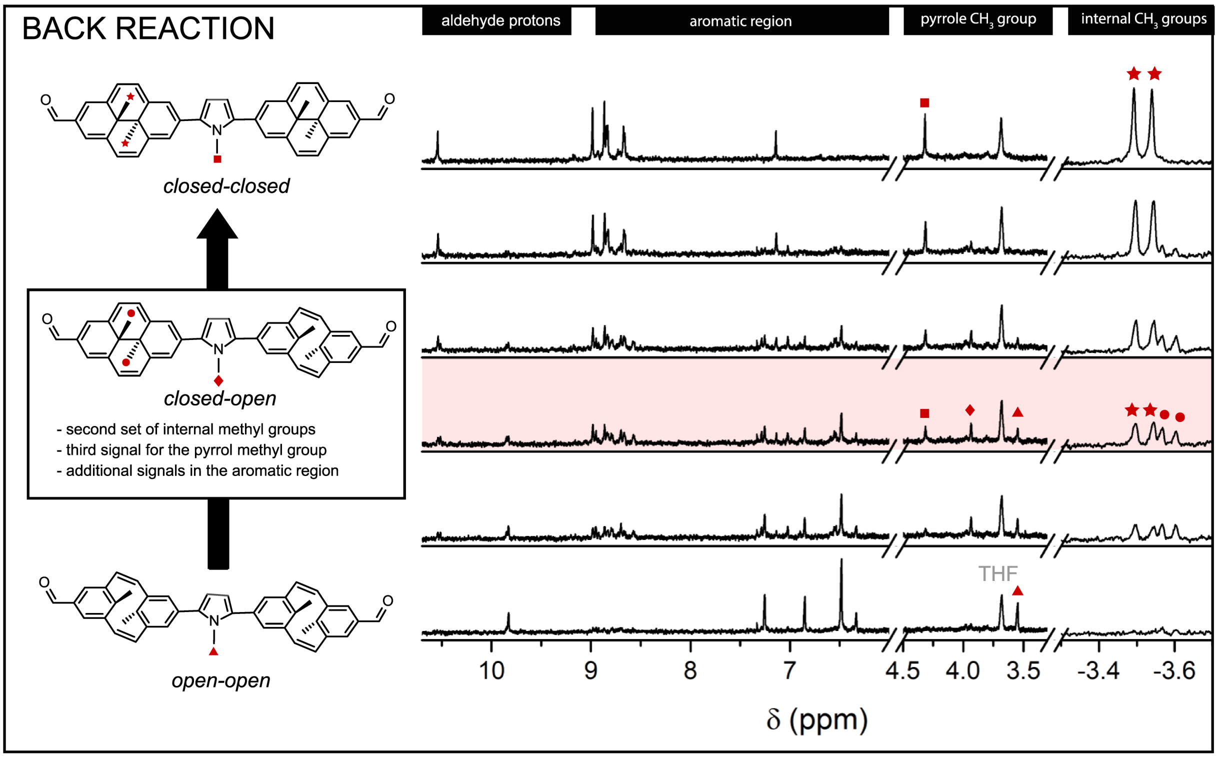

Figure S28. NMR-spectra of **PyFm-dimer** (CD_2Cl_2) after irradiation in the dark at 15 °C.

5. Quantum Yield Determination

5.1 PyFm-dimer

For the determination of both ring-opening quantum yields the **PyFm-dimer** was first pre-irradiated at 0 °C with 579 nm (intense 500 W mercury lamp) for 180 min. Then, irradiation with a 660 nm LED was performed to reach the photothermal equilibrium (PTE). The back reaction was monitored for 60 min in the dark. The cuvette was warmed up to room temperature and cooled back to 0 °C to verify the complete recovery of the **cc** isomer.

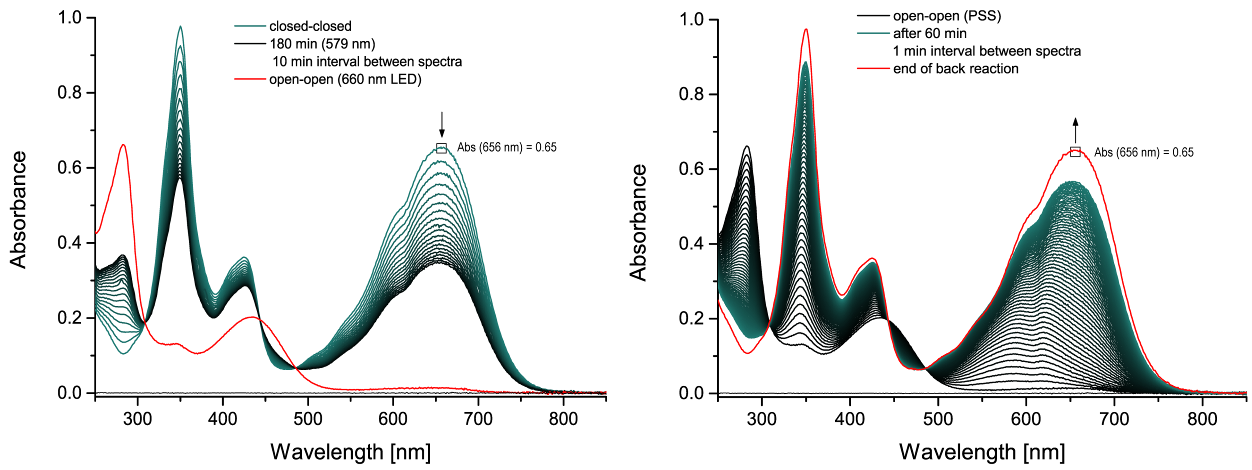

Figure S29. 660 nm irradiation (left) and thermal back reaction (right) of **PyFm-dimer** in THF (9.8 · 10^{-6} M) at 0 °C used for quantum yield determination.

The spectra of the thermal back reaction were divided by the pure **cc** spectra (Figure S30). In the wavelength region 700–730 nm the ratio appears constant, thus the **cc** isomer is the only isomer absorbing in that range.

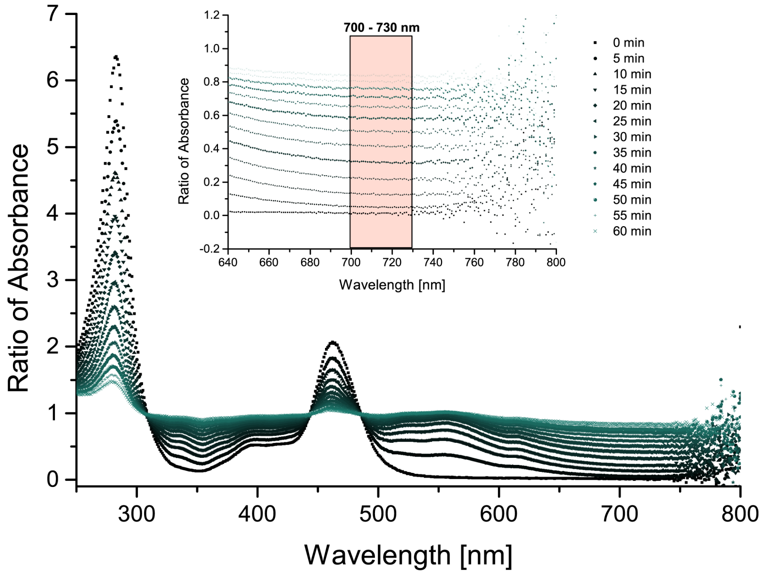

Figure S30. Spectra of thermal back reaction divided by spectra of pure **cc** isomer. In the region of 700–730 nm the ratio is constant and equals the amount of **cc** present at that time.

The average ratio equals the amount of **cc** isomer being built up with time during the thermal back reaction and the corresponding concentrations were fitted according to integrated rate laws for an irreversible consecutive reaction (Equation **I**, Figure S31). Because the reaction does not start with 100% **oo** isomer present, the remaining amount of **cc** was included in the fit, assuming the amount of **co** is negligible at the PTE.

$$cc(t) = 1 - \frac{k_2 \cdot e^{-k_1 \cdot t} - k_1 \cdot e^{-k_2 \cdot t}}{k_2 - k_1} + cc_0 \qquad \text{(I)}$$

$$oo(t) = e^{-k_1 \cdot t} - cc_0 \qquad \text{(II)}$$

$$co(t) = \frac{k_1}{k_2 - k_1} \cdot \left(e^{-k_1 \cdot t} - e^{-k_2 \cdot t}\right) \qquad \text{(III)}$$

cc (t)	amount of closed-closed isomer at time t	cc_0	amount of closed-closed isomer at start of reaction (t=0)
co (t)	amount of closed-open isomer at time t	$k_1 = k_{oo \to co}$	rate constant for first ring-closure
oo (t)	amount of open-open isomer at time t	$k_2 = k_{co \to cc}$	rate constant for second ring-closure

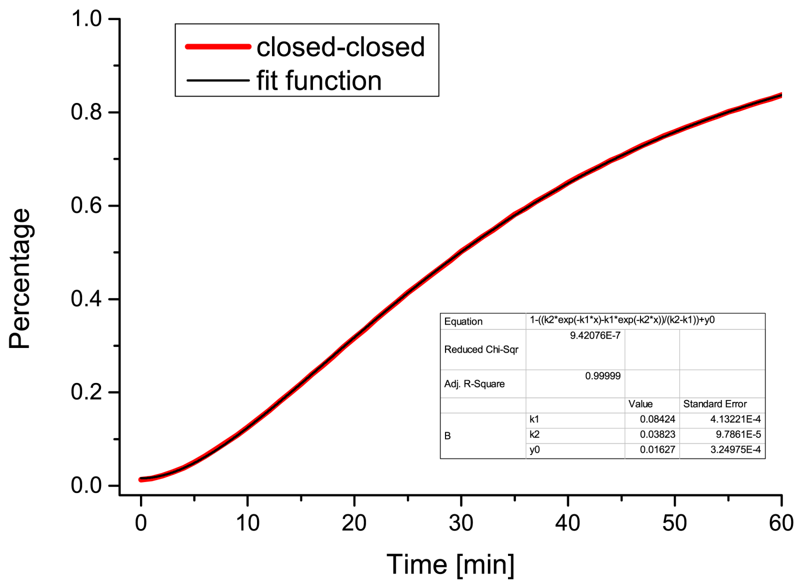

Figure S31. Fit of concentration of **cc** isomer during thermal back reaction according to Eq. I

The values for k_1 and k_2 allowed the determination of the concentrations of **oo** and **co** at any point of time during the back reaction using equation II and III, respectively (Figure S32).

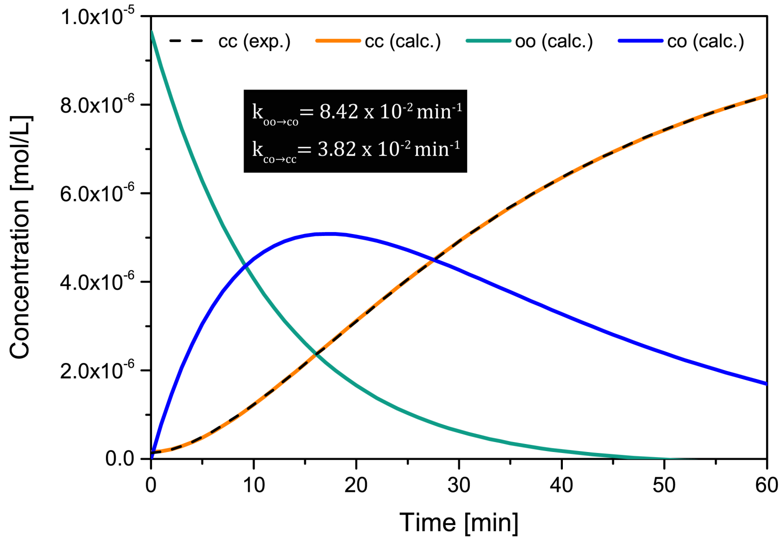

Figure S32. Concentrations of all three isomers during the thermal back reaction at 0 °C. The concentrations of **cc** were derived from the experimental spectra in order to calculate the rate constants. The concentrations of the other two isomers were calculated according to the kinetic rate laws for irreversible consecutive reactions.

Next, the spectra of pure **oo** and **co** isomer were calculated (see MS Figure 4a). At the photothermal equilibrium (*t* = 0 for the thermal back reaction) the **oo** isomer is the major isomer and the pure **oo** spectra was obtained by subtracting the amount of **cc** still present at the PTE (Equation IV). For the **co** isomer the spectra between 10 and 30 min were considered because in that time frame the **co** isomer is the predominating isomer and the amount of **cc** and **oo** at each time *t* were subtracted according to equation V.

$$A_\lambda(oo) = \frac{A_\lambda(PTE) - cc(PTE) \cdot A_\lambda(cc)}{1 - cc(PTE)} \qquad \text{(IV)}$$

$$A_\lambda(co) = \frac{A_\lambda(t) - cc(t) \cdot A_\lambda(cc) - oo(t) \cdot A_\lambda(oo)}{1 - cc(t) - oo(t)} \qquad \text{(V)}$$

$A_\lambda\,(oo)$	Absorbance at wavelength λ of pure oo isomer	$A_\lambda\,(co)$	Absorbance at wavelength λ of pure co isomer
$A_\lambda\,(cc)$	Absorbance at wavelength λ of pure cc isomer	$A_\lambda\,(t)$	Absorbance at wavelength λ at time t
$A_\lambda\,(PTE)$	Absorbance at wavelength λ at t=0	*oo (t)*	percentage of oo isomer present at time t
cc (PTE)	percentage of cc isomer present at t=0	*cc (t)*	percentage of cc isomer present at time t

With the calculated spectra, it was possible to determine the concentrations of each isomer during the irradiation. For the **cc** isomer the irradiation spectra are simply divided by the pure **cc** spectra and the obtained ratio in the range of 700–730 nm is used for determining the concentrations at the corresponding point of time. Subtraction of the amount of **cc** from the irradiation spectra and division by the pure **co** spectra identifies a constant region at 560–590 nm where the ratio equals the amount of **co** isomer present (Figure S33). The amount of **oo** isomer can then be obtained by the relationship *oo (t) = 1 – cc (t) – co (t)*.

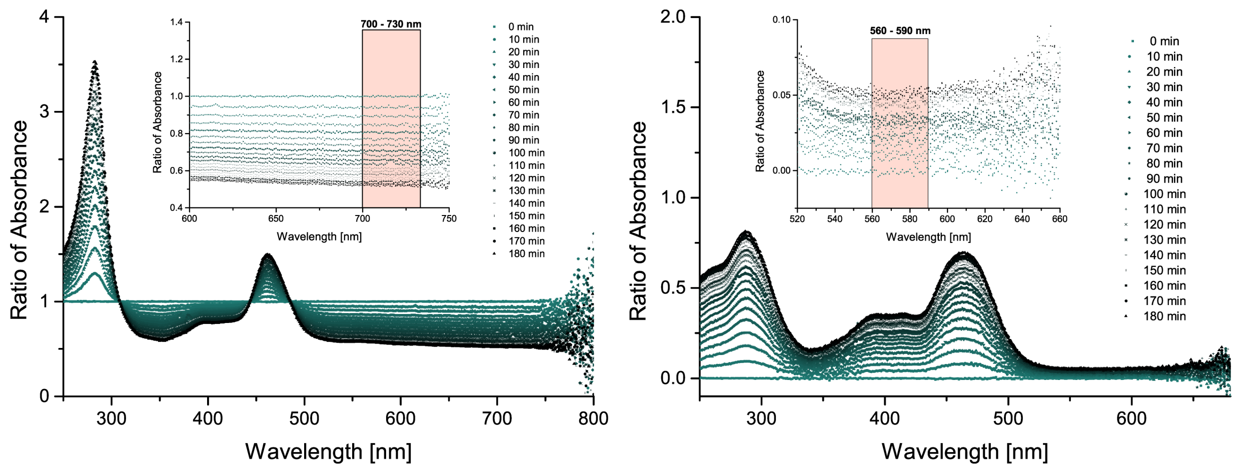

Figure S33. Division of irradiation spectra by the pure **cc** spectra shows a constant region at 700–730 nm (left). Irradiation spectra minus the amount of **cc** isomer, divided by pure **co** spectra (right) gives a constant region at 560–590 nm.

The differential equations for the concentrations of each isomer were numerically integrated and the previously determined concentrations were then fitted according to the following Equations (VI-VIII), providing values for $\phi_{cc\rightarrow co}$ and $\phi_{co\rightarrow oo}$ based on the least-square method (see Figure S34).

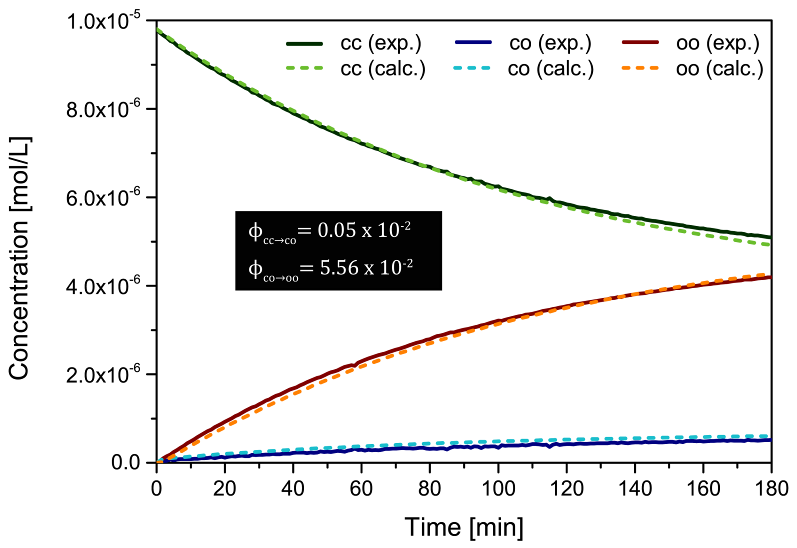

Figure S34. Comparison of the experimentally determined concentrations during irradiation and concentrations obtained by least-square fit method.

$$[cc]_{t+\Delta t} = [cc]_t + (-1000 \cdot I_0 \cdot F(t) \cdot d \cdot v^{-1} \cdot \phi_{cc\to co} \cdot \varepsilon_{cc} \cdot [cc]_t + k_{co\to cc}[co]_t) \cdot \Delta t \quad \text{(VI)}$$

$$[co]_{t+\Delta t} = [co]_t + (1000 \cdot I_0 \cdot F(t) \cdot d \cdot v^{-1} \cdot (\phi_{cc\to co} \cdot \varepsilon_{cc} \cdot [cc]_t - \phi_{co\to oo} \cdot \varepsilon_{co} \cdot [co]_t) - k_{co\to cc}[co]_t + k_{oo\to co}[oo]_t) \cdot \Delta t \quad \text{(VII)}$$

$$[oo]_{t+\Delta t} = [oo]_t + (1000 \cdot I_0 \cdot F(t) \cdot d \cdot v^{-1} \cdot \phi_{co\to oo} \cdot \varepsilon_{co} \cdot [co]_t - k_{oo\to co}[oo]_t) \cdot \Delta t \quad \text{(VIII)}$$

I_0	light intensity by actinometry	$[cc]_t$	concentration of cc isomer at time t
d	thickness of cuvette (1 cm)	$[co]_t$	concentration of co isomer at time t
v	volume of cuvette (3 cm^3)	$[oo]_t$	concentration of oo isomer at time t
Δt	time interval (1 min)	$\phi_{cc\to co}$	quantum yield for first ring-opening
$F(t)$	photokinetic factor	$\phi_{co\to oo}$	quantum yield for second ring-opening
ε_{cc}	extinction coefficient of cc isomer at 579 nm	$k_{oo\to co}$	rate constant for first ring-closure
ε_{co}	extinction coefficient of co isomer at 579 nm	$k_{co\to cc}$	rate constant for second ring-closure

5.2 PyFm-monomer

The determination of the quantum yield for the **PyFm-monomer** was conducted under the same conditions and with the same experimental setup as for the dimer. Irradiation with 579 nm (500 W mercury lamp) until the photothermal equilibrium, was followed by monitoring the thermal back reaction in the dark at 0 °C (Figure S35).

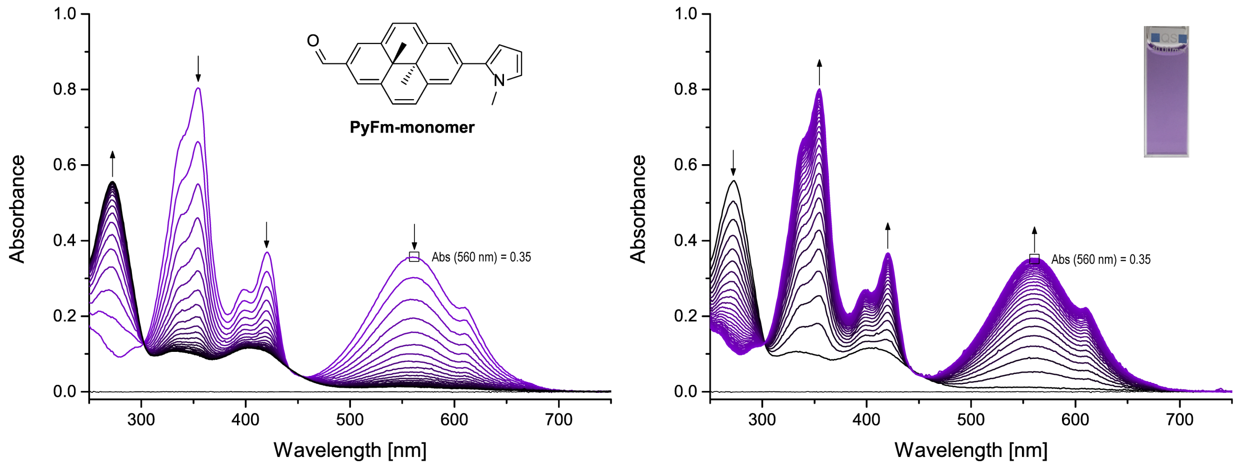

Figure S35. 579 nm irradiation (left, 15 s between consecutive spectra) and thermal back reaction (right, 5 min between consecutive spectra) of **PyFm-monomer** in THF (1.58 · 10^{-5} M) at 0 °C.

The rate constant for the thermal back reaction was obtained as $k_{o \rightarrow c}$ = 2.61 x 10^{-2} min^{-1} by plotting the change in absorbance at 579 nm against time (Figure S36), which corresponds to a half-life of approximately 27 min. The irradiation spectra were divided by the spectra of the pure closed form and in the region of 550–590 nm the ratio of the spectra corresponds to the amount of closed isomer present during the irradiation (Figure S37).

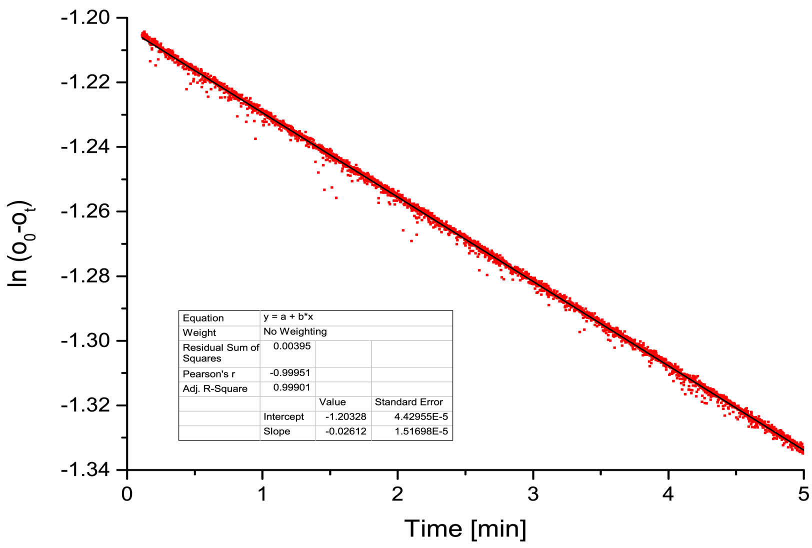

Figure S36. Fit of the change in absorbance for the thermal back reaction of **PyFm-monomer** to determine the rate constant.

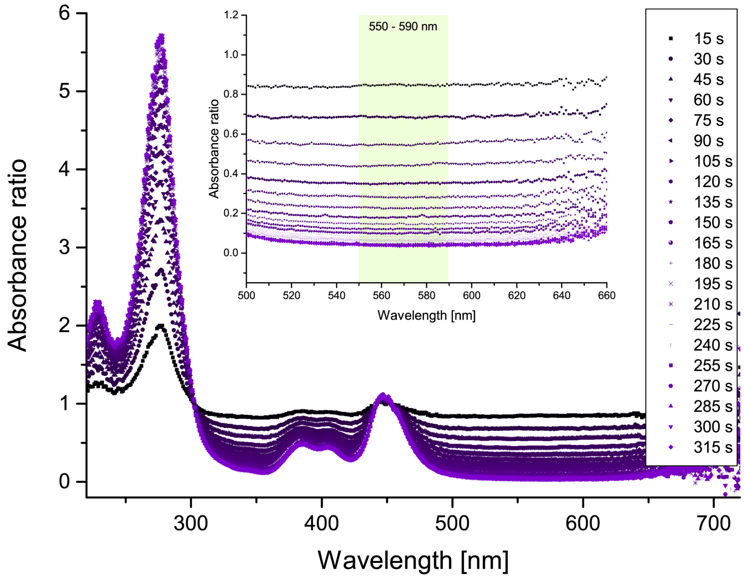

Figure S37. Division of irradiation spectra by the initial spectra of the pure closed isomer.

The quantum yield $\phi_{c \to o}$ = 9.90 x 10^{-2} is calculated based on Equation XI which is obtained by numerical integration of equation IX.

$$\frac{d[c](t)}{dt} = -I_0 \cdot F(t \cdot d \cdot v^{-1} \cdot \phi \cdot \varepsilon \cdot [c](t) + k \cdot [o](t) \qquad \text{(IX)}$$

$$[c]_{t+\Delta t} = [c]_t + \left(-1000 \cdot I_o \cdot d \cdot v^{-1} \cdot \frac{\left(1 - 10^{-Abs(t)}\right)}{Abs(t)} \cdot \phi \cdot \varepsilon \cdot [c]_t + k \cdot [o]_t\right) \cdot \Delta t \qquad \text{(X)}$$

$$\phi = \frac{\frac{[c]_{t+\Delta t}}{\Delta t} - k \cdot (c_0 - [c]_t)}{-1000 \cdot I_0 \cdot d \cdot v^{-1} \cdot \varepsilon \cdot \left(\frac{1 - 10^{-Abs(t)}}{Abs(t)}\right) \cdot [c]_t} \qquad \text{(XI)}$$

I_0	light intensity determined by actinometry	ε	extinction coefficient of c isomer at 579 nm
d	thickness of cuvette (1 cm)	$Abs_{579}(t)$	Absorbance at 579 nm at time t
v	volume of cuvette (3 cm^3)	$[c]_t$	concentration of c isomer at time t
k	rate constant for thermal ring-closure	$[o]_t$	concentration of o isomer at time t
Δt	time interval (0.25 min)	ϕ	quantum yield for ring-opening

6. Computational Methods

6.1. Methodology

For photochemical reactions involving higher excited states and their crossings, the methods of the choice are the Complete Active Space SCF (CASSCF) and CASPT2 that includes dynamic correlation energy in a perturbative approach. Previously Boggio-Pasqua *et al.*[8] studied the DHP to CPD transformation of the single unsubstituted chromophore with this methodology using active space of 16 electrons in 16 orbitals. Such calculations are still on the edge of current computational resources and would clearly yield a totally non-tractable active space for the dimers. Therefore, for the present investigation, we used Time-Dependent Density Functional Theory (TD-DFT) as computationally affordable choice. As was shown recently by Boggio-Pasqua and Garavelli[9] the TD-CAM-B3LYP[10] shapes of the potential energy surface of excited states (ES) are comparable with the one obtained with CAS/CASPT2. Moreover CAM-B3LYP is well-suited for charge transfer excitations which may occur as result of push-pull character of some of the investigated dimers. We first performed ground state (GS) optimizations with the CAM-B3LYP functional and the 6-31G(d) basis set, followed by vibrational frequency calculation. Optimizations of excited state were performed at the same level of theory. The influence of the solvent (chloroform) was included by means of Polarizable Continuum Model (PCM). For ES calculations, the linear response formalism (LR)[11] of PCM was used. For all of our calculations the Gaussian16 program[12] was used with the so-called *ultrafine* DFT integration grid and a tight SCF-KS convergence criterion.

6.2 Solvent effects on molecular structure

In the experiment different solvents were used for different molecules to allow good solubility at given conditions (THF for **PyFm-dimer** and DCM for **PhCN-dimer**, again THF for **Ester-dimer**). In the prediction phase of the experimental work we used gas phase geometry optimizations to avoid any confusion regarding the choice of solvent. We observed several situations with oscillator strength borrowing between states in gas phase and thus the ring-opening state was identified mainly based on orbital picture and not on the basis of the computed f. The optimized geometries are usually very similar in gas phase and the solvent (differences of ca. 0.002 Å) with one interesting exception. We have found that the twisting between DHP units of the **PyFm-dimer** responds significantly to solvent polarity. In the gas phase we observed q = 1.566 Å and in chloroform q = 1.549 Å. Smaller q relates to more conjugated (and parallel) DHP units in more polar solvents. Interestingly, this was not found for the structurally similar **PyCN-dimer** which attains planar DHP-bridge-DHP geometry already in the gas phase (with q very similar to chloroform). Both molecules have polar acceptor groups on the opposite side of the dimer. Repolarization of the formyl group in more polar solvents is likely the cause of the observed effect.

6.3 Vertical excitation energies

Table S1. Vertical excitation energies (VEE) and oscillator strengths (f) of the lowest 8 excited states of the DHP and DHP dimers calculated in chloroform**.**

Molecule	VEE [eV]	f [a.u]
**DHP**	2.3460	0.0061
	2.6517	0.0477
	3.3561	0.4513
	3.6865	1.2444
**PyFm-dimer**	2.1817	0.9416
	2.2756	0.0190
	2.3371	0.1333
	2.4218	0.0118
	3.1949	0.2669
	3.1965	0.2321
	3.3556	0.7029
	3.4403	0.4565
**PhCN-dimer**	2.1705	1.0571
	2.2433	0.2246
	2.3279	0.0015
	2.4132	0.1459
	3.1504	0.3217
	3.2048	1.2413
	3.2440	0.4466
	3.4625	0.3561
**Ester-dimer**	2.4167	0.0047
	2.4169	0.0039
	2.5445	0.4100
	2.6191	0.0071
	3.3596	0.3461
	3.3761	0.3785
	3.5187	1.0815
	3.6865	0.0138
**CN-dimer**	1.9936	1.5307
	2.2089	0.0000
	2.2167	0.0269
	2.3944	0.0000
	3.1154	0.0000
	3.1203	0.5379
	3.2462	0.0000
	3.3205	1.6190
**Isoin-dimer**	2.2562	1.2311
	2.2721	0.1647
	2.2907	0.0958
	2.4476	0.0064
	2.7596	0.2060
	2.9799	0.0019
	3.2341	0.2084
	3.2410	0.2618
**PyCN-dimer**	1.8933	1.8320
	2.0741	0.0268
	2.1448	0.0832
	2.2081	0.0323
	3.0239	0.0692
	3.0459	0.2208
	3.0712	0.1824
	3.1470	0.2636

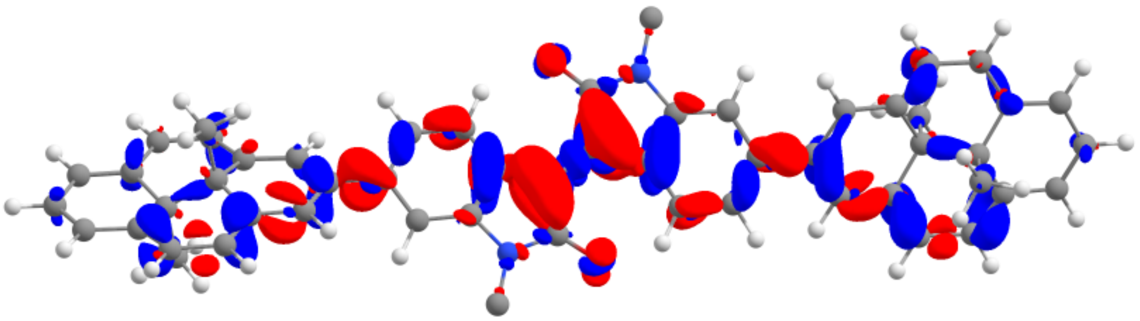

Figure S38. Electron density differences upon the excitation to the S_1 state of the **Isoin-dimer**. The red and blue lobes indicate regions of increase and decrease of electron density upon excitation. Side alkyl chains were omitted for clarity. Isosurface value 0.001 au.

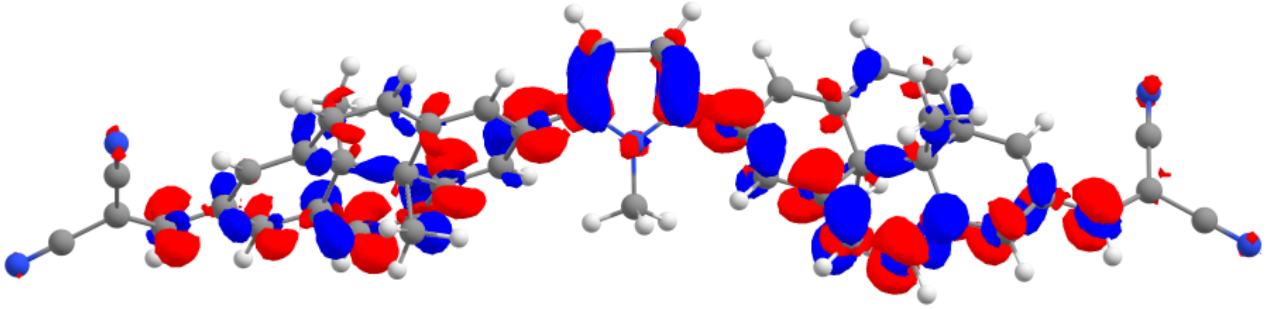

Figure S39. Electron density differences upon the excitation to the S_1 state of the **PyCN-dimer**. The red and blue lobes indicate regions of increase and decrease of electron density upon excitation. Isosurface value 0.001 au.

6.4 Adiabatic excitation energies

Table S2. Adiabatic excitation energies (AEE) of the DHP and DHP dimers calculated in chloroform. All values are in eV.

Molecule	State	AEE [eV]
**DHP**	S_2	2.0634
**PyFm-dimer**	S_1	1.5463
**PhCN-dimer**	S_1	1.6054
**Ester-dimer**	S_3	1.8420
**CN-dimer**	S_1	1.4481
**Isoin-dimer**	S_1	1.5867
**PyCN-dimer**	S_1	1.2970

7. References

[1] R. H. Mitchell, V. Boekelheide, *J. Am. Chem. Soc.* 1974, *96*, 1547–1557.

[2] R. H. Mitchell, Y.-H. Lai, R. V. Williams*, J. Org. Chem.* **1979**, *44*, 4733–4735.

[3] S. S. Dharmapurikar, A. Arulkashmir, C. Das, P. Muddellu and K. Krishnamoorthy, *ACS Appl. Mater. Interfaces* **2013,** *5*, 7086–7093.

[4] L. Cai, T. Moehl, S.-J. Moon, J.-D. Decoppet, R. Humphry-Baker, Z. Xue, L. Bin, S. M. Zakeeruddin, M. Grätzel, *Org. Lett.* **2014,** *16*, 106–109.

[5] K. Klaue, W. Han, P. Liesfeld, F. Berger, Y. Garmshausen, S. Hecht, *J. Am. Chem. Soc.* DOI: 10.1021/jacs.0c04219.

[6] R. H. Mitchell. D. Y. K. Lau, A. Miyazawa, *Org. Prep. Proceed. Int.* **1996**, *28*, 713–716.

[7] T. Sumi, Y. Takagi, A. Yagi, M. Morimoto, M. Irie, *Chem. Commun.* **2014**, *50*, 3928–3930.

[8] M. Boggio-Pasqua, J. M. Bearpark, A. M. Robb, *J. Org. Chem.* **2007**, 72, 4497-4503.

[9] M. Boggio-Pasqua, M. Garavelli, *J. Phys. Chem. A*, **2015**, 119, 6024-6032.

[10] T. Yanai, D. P. Tew, N. C. Handy, *Chem. Phys. Lett.,* **2014**, 393, 51–57.

[11] R. Cammi, B. Mennucci, *J. Chem. Phys.,* **1999**, 110, 9877–9886.

[12] M. J. Frisch et al. Gaussian 16 Revision A.03, **2016**, Gaussian Inc. Wallingford CT.
